# Notch-dependent DNA *cis*-regulatory elements and their dose-dependent control of *C. elegans* stem cell self-renewal

**DOI:** 10.1242/dev.200332

**Published:** 2022-04-08

**Authors:** Tina R. Lynch, Mingyu Xue, Cazza W. Czerniak, ChangHwan Lee, Judith Kimble

**Affiliations:** 1 Department of Biochemistry, University of Wisconsin-Madison, Madison, WI 53706, USA; 2 Integrated Program in Biochemistry, Madison, WI 53706, USA; 3 Department of Life Sciences, Imperial College London, South Kensington, London, SW7 2AZ, UK; 4 Joint Department of Biomedical Engineering, Marquette University and Medical College of Wisconsin, Milwaukee, WI 53226, USA; 5 Department of Biological Sciences, University at Albany, State University of New York, Albany, NY 12222, USA

**Keywords:** Gradient, Homotypic cluster, smFISH, Spatiotemporal resolution, *sygl-1*, Transcription factor binding site

## Abstract

A long-standing biological question is how DNA *cis*-regulatory elements shape transcriptional patterns during metazoan development. Reporter constructs, cell culture assays and computational modeling have made major contributions to answering this question, but analysis of elements in their natural context is an important complement. Here, we mutate Notch-dependent LAG-1 binding sites (LBSs) in the endogenous *Caenorhabditis elegans sygl-1* gene, which encodes a key stem cell regulator, and analyze the consequences on *sygl-1* expression (nascent transcripts, mRNA, protein) and stem cell maintenance. Mutation of one LBS in a three-element cluster approximately halved both expression and stem cell pool size, whereas mutation of two LBSs essentially abolished them. Heterozygous LBS mutant clusters provided intermediate values. Our results lead to two major conclusions. First, both LBS number and configuration impact cluster activity: LBSs act additively in *trans* and synergistically in *cis*. Second, the SYGL-1 gradient promotes self-renewal above its functional threshold and triggers differentiation below the threshold. Our approach of coupling CRISPR/Cas9 LBS mutations with effects on both molecular and biological readouts establishes a powerful model for *in vivo* analyses of DNA *cis-*regulatory elements.

## Abstract (precis)

**Summary:** Notch-dependent DNA *cis*-regulatory elements work together in their developmental context in *C. elegans* to shape a transcriptional gradient, control stem cell pool size, and govern differentiation onset.

## INTRODUCTION

Cell signaling and transcriptional regulation are central to metazoan development. Though signaling pathways vary tremendously, a common theme is their patterned control of gene expression via DNA *cis-*regulatory elements (CREs). Traditional analyses of signaling have relied on manipulations of the pathway's core components (e.g. Austin and Kimble, 1987; Beumer and Clevers, 2021; Lee et al., 2016). Such pathway-level intervention has been powerful, but likely impacts multiple target genes and circuits. An alternative approach manipulates signal-dependent CREs. Clusters of closely spaced CREs are common in animal genomes, and CRE point mutations are crucial in evolution and disease (Crocker et al., 2016; Ezer et al., 2014; Gotea et al., 2010; Madani Tonekaboni et al., 2019; Melton et al., 2015; Payne and Wagner, 2015; Stern and Orgogozo, 2009). Metazoan CREs have been subjected to many elegant analyses, in classic assays with reporter transgenes and heterologous cells, and increasingly with integration of empirical data and computational modeling (e.g. Andersson and Sandelin, 2020; Bentovim et al., 2017; Ezer et al., 2014; Giorgetti et al., 2010; Hardison and Taylor, 2012; Lammers et al., 2020; Shlyueva et al., 2014; Wong and Gunawardena, 2020). The advent of CRISPR/Cas9 gene editing has now made metazoan CREs accessible in their natural developmental context. The analysis of CREs in endogenous genes has the potential to solidify principles gleaned from classic, yet more artificial, experiments and also to advance our understanding of how *cis*-regulatory elements function during development.

**Fig. 1.**
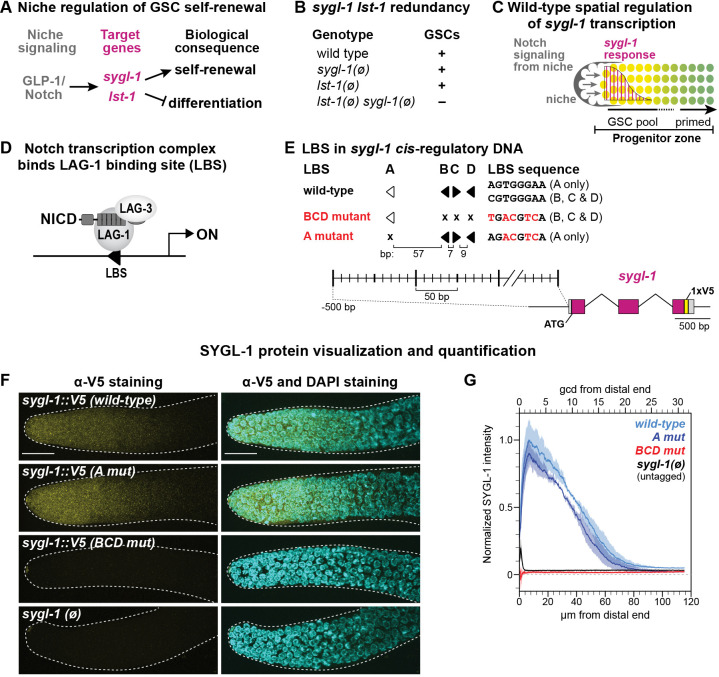
**Identification of functional LBSs in *sygl-1 cis*-regulatory DNA.** (A) *C. elegans* GSC molecular regulators. (B) GSCs are maintained in null mutants (ø) of either *sygl-1* or *lst-1,* but not maintained when both genes are null. (C) Schematic of the distal gonad. The niche (gray) is a somatic cell at the distal end. GLP-1/Notch signaling (arrows) maintains a pool of GSCs in the distal PZ (yellow) and activates graded *sygl-1* transcription (magenta). Germ cells in the proximal PZ become primed for differentiation (green). (D) The Notch transcriptional activation complex binds DNA (black line) at an LBS (black arrowhead) via its CSL DNA-binding protein (LAG-1 in *C. elegans*). Other components are the Notch intracellular domain (NICD) and Mastermind-like coactivator (LAG-3 in *C. elegans*). Right-facing arrow labeled ‘ON’ represents predicted transcription start site. (E) Summary of the LBS mutations. Filled arrowheads represent canonical 5′-YGTGRGAA-3′ LBS; open arrowheads represent noncanonical LBSs. Arrowheads point right for 5′-CGTGGGAA-3′ and left for its complement 5′-TTCCCACG-3′. Mutant LBS (x) is 5′-TGACGTCA-3′ for LBS B, C and D, and 5′-AGACGTCA-3′ for LBS A (mutated bases underlined, or in red in figure). Spacing in base pairs (bp) between LBS is to scale. 1xV5 (yellow) was inserted to visualize SYGL-1 protein. Exons are shown in magenta and untranslated regions (UTRs) in gray. (F) Representative maximum-intensity *z*-projections of V5-stained dissected distal gonads. Scale bar: 20 µm. For all figures throughout this paper, see Table S1 for strain genotypes*.* (G) Fiji quantification of V5 immunosignal normalized to *sygl-1::V5(wt)* (see Materials and Methods). Germ cell position measures are germ cell diameters (gcd; top axis) and microns (µm; bottom axis). Total gonads scored from two independent experiments: wild type, 29; *A mut*, 31; *BCD mut*, 25; *sygl-1(ø)*, 24.

Here, we take advantage of a well-established model system to investigate how a homotypic cluster of CREs controls stem cells *in vivo*. We focus on Notch-dependent CREs in the *C. elegans sygl-1* gene, which encodes a key regulator of self-renewal in germline stem cells (GSCs) (reviewed by Hubbard and Schedl, 2019). In this small nematode, GLP-1/Notch signaling from the niche activates transcription of two target genes, *sygl-1* and *lst-1* (Kershner et al., 2014; Lee et al., 2016; Shin et al., 2017) (Fig. 1A). These genes are functionally redundant: either *sygl-1* or *lst-1* can maintain GSCs on its own, but removal of both genes triggers premature differentiation and loss of GSCs, the Glp phenotype (Fig. 1B) (Kershner et al., 2014). Indeed, *sygl-1* and *lst-1* are likely the only Notch targets responsible for GSC maintenance (Chen et al., 2020). We previously reported that *sygl-1* and *lst-1* transcription is graded in germ cells within the niche (Fig. 1C) (Kershner et al., 2014; Lee et al., 2016), and that SYGL-1 and LST-1 proteins are restricted to a distal region within the progenitor zone (PZ), where GSCs reside (Fig. 1C, yellow) (Haupt et al., 2019; Shin et al., 2017). When SYGL-1 expression is manipulated to expand, the GSC pool correspondingly expands, suggesting that the spatial extent of SYGL-1 protein determines where GSC daughters transition from a stem cell state to one primed for differentiation (Shin et al., 2017). However, only *sygl-1* null mutants were available before this work, so the impact of reduced *sygl-1* was unknown.

The *sygl-1* gene is well poised for CRE mutational analyses. Its transcription in GSCs relies on signaling by a single pathway, the Notch pathway (Kershner et al., 2014), and its wild-type transcriptional response to Notch signaling has been described at high resolution (Lee et al., 2016). Notch activates transcription via a conserved protein complex that includes LAG-1, a member of the CSL [CBF1-RBPJκ/Su(H)/LAG-1] family of DNA-binding proteins; the intracellular domain of the Notch receptor (NICD); and the LAG-3 (also known as SEL-8) Mastermind-like coactivator (Fig. 1D) (reviewed by Greenwald and Kovall, 2013). LAG-1 anchors the complex to DNA at LAG-1 binding sites (LBSs), motifs conserved for CSL proteins across animal phylogeny (Christensen et al., 1996; Nellesen et al., 1999; Tun et al., 1994). The *sygl-1* 5′ flanking region possesses four computationally predicted LBSs that are covered by a LAG-1 ChIP-seq peak (Fig. 1E) (Chen et al., 2020; Yoo et al., 2004). Moreover, a 1 kb *sygl-1* DNA fragment harboring those LBSs drives GFP reporter expression in GSCs (Kershner et al., 2014). Wild-type *sygl-1* transcripts are graded across the GSC pool and become low or undetectable as GSCs are triggered to begin differentiation (Lee et al., 2016) (Fig. 1C). SYGL-1 protein, visualized with a V5 epitope tag, is patterned similarly to *sygl-1* RNA (Shin et al., 2017). As oocytes begin to mature in the proximal germline, *sygl-1* transcription occurs again, this time independently of Notch signaling (Kershner et al., 2014; Lee et al., 2016). Previous analyses of Notch-dependent *sygl-1* transcription laid an important foundation for our investigation of *sygl-1* CRE mutations. Single-molecule fluorescence *in situ* hybridization (smFISH) in fixed wild-type gonads identified a gradient in the probability of Notch-dependent transcriptional activation, scored as the percentage of cells with nuclear active transcription sites (ATSs), and a similarly shaped gradient in the accumulation of cytoplasmic mRNAs (Lee et al., 2016). A partial loss-of-function GLP-1/Notch receptor generated shallower gradients, linking Notch signaling strength to the shape of graded *sygl-1* expression. Live imaging extended the smFISH results to reveal that the strength of Notch signaling corresponds to the duration of a *sygl-1* transcriptional burst (Lee et al., 2019), a result confirmed by a parallel study in *Drosophila* embryos (Falo-Sanjuan et al., 2019).

Our approach to *in vivo* investigations of CRE function takes advantage of a metazoan exemplary for its relative simplicity and tractability. Multiple and diverse CREs have been implicated in regulation of transcriptional patterns during development, which can limit the interpretability of CRE mutation experiments (Kuang et al., 2021; Li et al., 2021; Park et al., 2019). However, tackling CRE function in a well-defined model such as *Caenorhabditis elegans*, and the *sygl-1* gene in particular, strips layers of complexity. For example, in flies and mammals, a cooperative physical interaction between neighboring NICD proteins promotes transcriptional synergy when CSL binding sites are spaced ∼15-17 bp apart and arranged with head-to-head polarity (Arnett et al., 2010; Bailey and Posakony, 1995; Cave et al., 2005; Kobia et al., 2020; Kuang et al., 2021; Nam et al., 2007; Nellesen et al., 1999; Severson et al., 2017). This synergy relies on a salt bridge between a lysine in one NICD and a glutamate in the second NICD. However, that molecular interface is not conserved in nematode NICD (Nam et al., 2007), and disruption of head-to-head LBSs did not affect reporter expression in *C. elegans* embryos (Neves et al., 2007). Although *C. elegans* NICDs may interact by some other means, lack of the salt bridge found in flies and mammals removes one layer of complexity. Regardless, study of a tractable *in vivo cis*-regulatory module may reveal principles relevant to homotypic clusters more broadly.

Here, we couple Cas9 gene editing with quantitative *in vivo* analyses to investigate functions of individual *sygl-1* LBSs within a homotypic cluster. LBS number emerges as a major force in shaping the developmental gradient: LBS dose modulates the probability and intensity of *sygl-1* transcription, the abundance of *sygl-1* mRNA and protein, and the size of the GSC pool. Furthermore, *sygl-1* LBSs in *trans*, residing on opposite homologous chromosomes, act additively, whereas *sygl-1* LBSs in *cis*, residing on the same chromosome, act synergistically. Finally, we identify rough boundaries for the threshold of SYGL-1 abundance required for self-renewal. This *in vivo* investigation provides a model for learning how DNA *cis*-regulatory elements transform signaling inputs into reproducible patterns of gene expression during development.

## RESULTS

### A cluster of three *sygl-1* LBSs activates Notch-dependent expression

Four computationally predicted Notch-dependent *cis*-regulatory elements are named LBS A-D (Yoo et al., 2004) (Fig. 1E). LBS B, C and D exist in a cluster whereas LBS A resides upstream of the cluster. In other *Caenorhabditis* species, *sygl-1* 5′ flanking regions contain LBS clusters in species-specific patterns with at least two LBSs (Fig. S1A). LBS B, C and D sequences adhere to the canonical CSL binding motif, whereas LBS A lacks the initial pyrimidine and is thus noncanonical. Such noncanonical LBSs compete poorly for CSL binding in gel-shift assays (Christensen et al., 1996; Nellesen et al., 1999; Torella et al., 2014; Tun et al., 1994), and are expected to be weaker sites *in vivo*.

We hypothesized that the BCD cluster is largely responsible for Notch-dependent regulation of *sygl-1* expression in GSCs. To test this idea, we used Cas9 gene editing to generate two mutants in endogenous *sygl-1* DNA: in *A mut*, four base pairs are changed in LBS A, and in *BCD mut* five base pairs are changed in each of LBS B, C and D (Fig. 1E, see Materials and Methods mutant design). To visualize SYGL-1 protein, we inserted a V5 epitope tag in each mutant (Fig. 1E); this V5 tag has no detectable effect on SYGL-1 protein function (Shin et al., 2017). Initial analyses focused on the distal gonad, where *sygl-1* expression is Notch dependent, and were performed in animals with wild-type *lst-1* to ensure a healthy germline. *A mut* had a wild-type pattern of SYGL-1 protein, both in abundance and distribution, and thus mutation of LBS A had no apparent effect (Fig. 1F,G). By contrast, *BCD mut* made no SYGL-1 and thus the pattern of SYGL-1 protein was lost (Fig. 1F,G). In the presence of wild-type *lst-1*, the functionally redundant counterpart of *sygl-1*, *BCD mut* animals were fertile and their germlines were organized normally. However, upon *lst-1* removal, *BCD mut* adults were sterile with a Glp phenotype (loss of GSCs) (Fig. S1B). Therefore, the *BCD mut* germline defect was similar to *sygl-1(ø)*. However, unlike *sygl-1(ø)*, *BCD mut* animals expressed SYGL-1 in the proximal germline (Fig. S1C), where it is Notch independent (Kershner et al., 2014) (see Introduction). Proximal SYGL-1 protein was also present in *A mut* gonads (Fig. S1C). The *BCD mut* lack of distal SYGL-1 and inability to support GSC self-renewal are consistent with the idea that LBSs drive Notch-specific *sygl-1* expression in the distal gonad. In an attempt to increase expression, we transformed LBS A to a canonical motif, but found no effect (Fig. S1D,E). We conclude that the BCD cluster is primarily responsible for Notch regulation of SYGL-1 expression in GSCs.

**Fig. 2.**
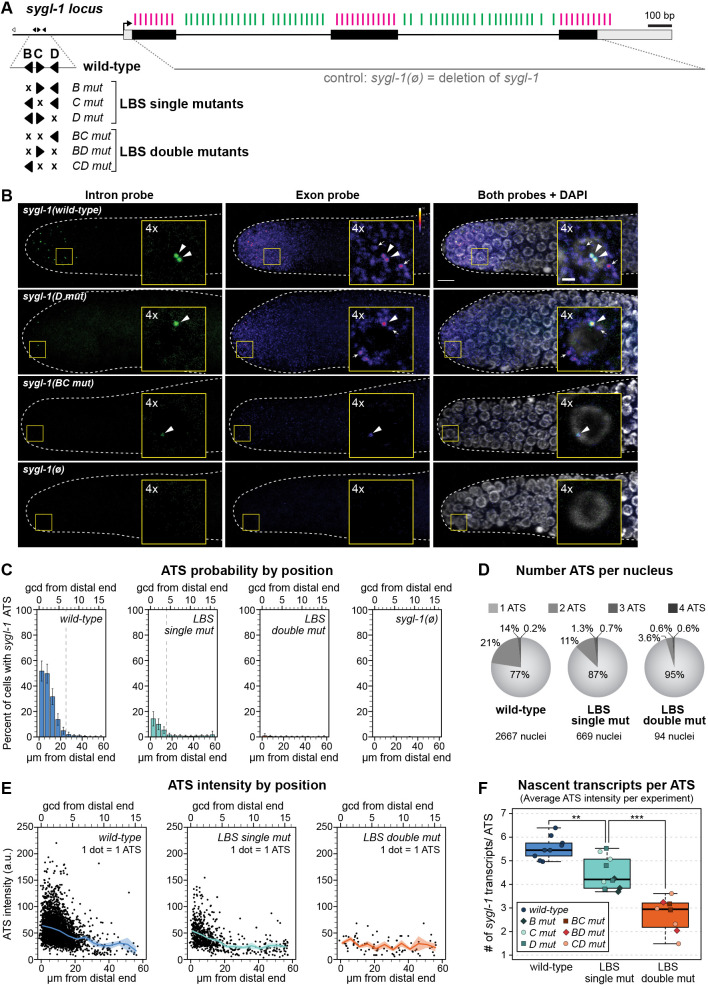
**LBS mutations weaken niche-dependent transcriptional response of *sygl-1.*** (A) Schematic of the *sygl-1* locus. Black boxes represent exons, gray boxes represent UTRs. Black arrow indicates predicted transcription start site. smFISH probes are shown above DNA as vertical lines: exon probes (magenta) and intron probes (green) (see Materials and Methods). The *sygl-1(ø)* control removes the DNA sequence that probes detect. Expansion shows individual LBSs and their mutations; conventions as in Fig. 1E. LBS mutants in this figure are not epitope-tagged. (B) Representative images of *sygl-1* smFISH in the distal end of dissected adult gonads. Exon channel colors reflect intensity to show mRNA without saturating ATS (see Materials and Methods; color key in upper right; pixel intensities 3-50). DAPI shown in grayscale. Main images are maximum-intensity *z*-projections; insets are 4×-magnified maximum-intensity projections (four 0.3 µm slices). Arrowheads indicate ATS (overlapping intron/exon/DAPI). Arrows indicate cytoplasmic mRNA (exon only). Scale bars: 5 μm (main image); 1 μm (insets). (C-F) See Fig. S2E for specific values. (C) Percentage of nuclei with at least one ATS as a function of distance (units as in Fig. 1G). Vertical dashed line indicates transcriptional extent of gradient, after which <5% cells contain at least one ATS. Error bars represent s.e.m. (D) Pie charts showing the number of ATSs per nucleus for wild type and LBS mutants. Percentages are averages from three experiments (see Materials and Methods). Nuclei with zero *sygl-1* ATSs have been excluded. (E) Position of ATSs in wild type and single and double LBS mutants. Each dot represents one ATS (see Materials and Methods); see Fig. S2D for details (e.g. total ATSs scored). Solid line represents mean (5 µm intervals); shaded area represents s.e.m. (F) ATS intensities as a rough estimate of the number of nascent transcripts/ATS (see Materials and Methods). Boxplot center lines represent medians (wild type: 5.1; single mutants; 3.7; double mutants: 2.6); see Materials and Methods for BoxPlotR conventions. Each dot represents mean of all ATS/experiment (irrespective of position). Total number of experiments: wild type, 9; LBS single mutants, 10; LBS double mutants: 8. Experiments with zero ATS detected (*BC mut*, *BD mut*) are not represented. **P=0.0009; ***P=7.1×10^−5^ (Student's *t*-test).

**Fig. 3.**
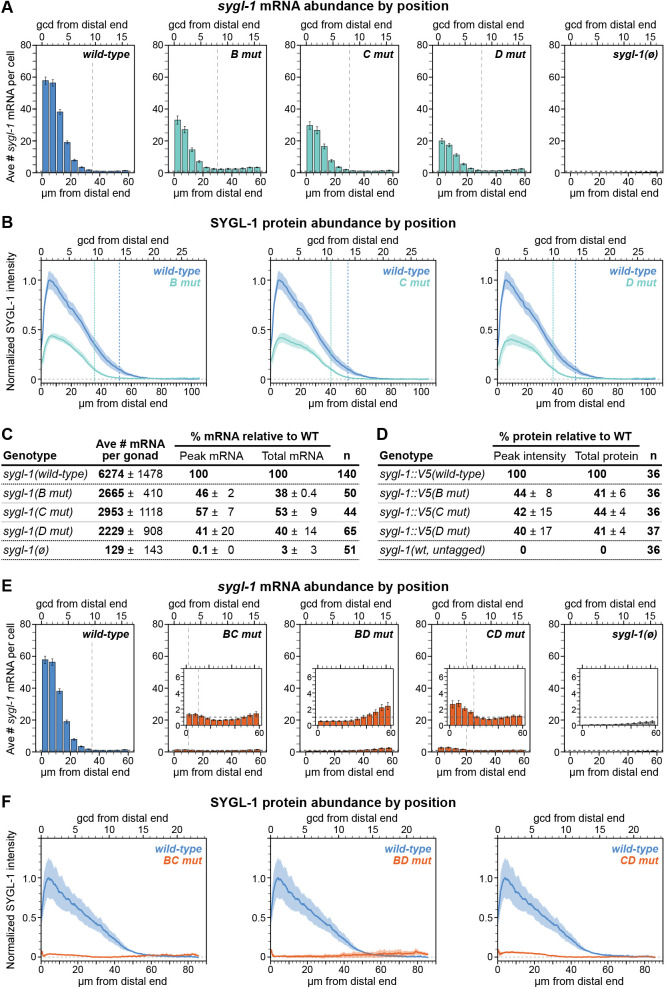
**LBS mutations reduce molecular *sygl-1* mRNA and protein abundance.** (A-F) Molecular output of LBS single (A-D) and double (E,F) mutants. (A,E) Average number of *sygl-1* mRNA molecules per cell (see Materials and Methods). See Fig. S2E for specific values. Position measures as in Fig. 1G. Error bars represent s.e.m. Vertical dashed lines indicate the extent of mRNA gradient after which values fall below background and/or reach minimum. Insets in E show the data near background in greater detail with horizontal dashed lines indicating the background level [from *sygl-1(ø)*; see Materials and Methods]. (B,F) Quantification of V5 immunofluorescence as a measure of SYGL-1 expression (see Materials and Methods). Solid lines represent mean; shaded area represents s.e.m. Horizontal dashed line at ‘0’ is based on an untagged control. Small peaks 0-3 µm from the distal end are nonspecific V5 signal (see Materials and Methods). Vertical dashed lines indicate the extent of the protein gradient after which values fall below 10% of the wild-type maximum. See D for total number of gonads scored in B; total gonads scored in F are from three or four experiments: wild type, 61; *BC mut*, 49; *BD mut*, 59 *CD mut*, 39. (C) Summary of mRNA data in LBS single mutants. Numbers are mean per experiment ±s.d. between experiments (see Materials and Methods). *n*, number of gonads scored in at least three experiments. Peak mRNA is the number of mRNA molecules per cell from the 0-5 µm region as a percentage of wild type; total mRNA is the number of mRNA molecules per gonad as a percentage of wild type. (D) Summary of protein data in LBS single mutants. Numbers are mean percentage of wild type for each replicate ±s.d. between replicates (see Materials and Methods). *n*: total gonads scored in three replicates. All data were obtained in adults carrying *lst-1(+)*; RNA data (A,C,E) is from smFISH experiments (strains not epitope-tagged) and protein data from V5 immunostaining (B,D,F).

**Fig. 4.**
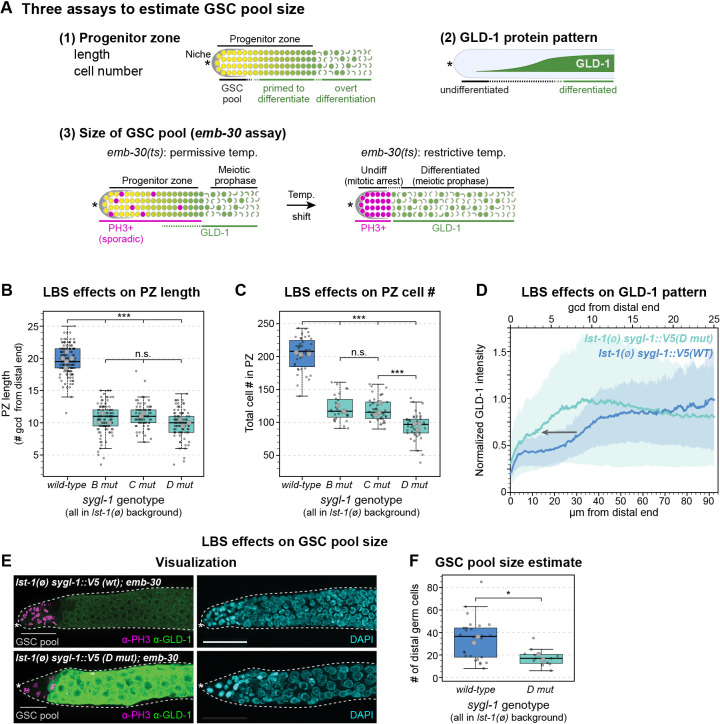
**LBS mutations reduce GSC pool size.** (A) Schematics representing three complementary assays used to estimate GSC pool size. Top left: PZ with progression from GSCs (yellow) to overt differentiation (green; meiotic prophase). Top right: GLD-1 protein increases as germ cells differentiate. Bottom left: At 15°C, *lst-1(ø) sygl-1(x); emb-30(ts)* PZ contains scattered M-phase (PH3^+^, magenta) cells and increasing GLD-1 (green). Bottom right: When shifted to 25°C, PZ germ cells arrest. Distal PH3^+^, GLD-1^−^ cells are inferred to come from GSCs and proximal PH3^−^ cells; GLD-1^+^ cells are inferred to come from GSC daughters primed to differentiate. Asterisk indicates the distal end. (B,C) Boxplots showing the effects of LBSs on PZ length and cell number. Boxplot conventions match Fig. 2F. Small circles indicate individual gonads; large circles indicate replicate averages. Data from each gonad were fitted to a linear mixed effects model; Tukey's post-hoc test was used to make pairwise comparisons between genotypes. ***P<0.0001; n.s., not significant (*P*≥0.01). (B) Average number of germ cell diameters (gcd) in PZ were manually counted. Total gonads were scored in two to five experiments: wild type, 141; *B mut*, 106; *C mut*, 73; *D mut*, 97. (C) Total number of cells in the PZ were counted with Imaris software (see Materials and Methods). Total gonads were scored in three independent experiments: wild type, 40; *B mut*, 28; *C mut*, 42; *D mut*, 42. (D) Fixed gonads were stained with polyclonal antibody to GLD-1 to determine the effect of LBSs on GLD-1 expression. Arrow indicates the distal shift of the increase in GLD-1 expression. Position measures as in Fig. 1G. Signal intensities were normalized against internal controls (see Materials and Methods). Solid lines represent mean; shaded areas represent s.e.m.; 24 gonads/genotype scored from two replicates. (E) Representative maximum-intensity projections. Left: PH3 (magenta), GLD-1 (green). Gray lines indicate the extent of the GSC pool. Right: DAPI (cyan). Scale bar: 20 µm. (F) Estimated number of GSCs in the naïve pool; GLD-1^−^ PH3^+^ cells were manually counted (see Materials and Methods). Boxplot conventions as in B,C. Number of total gonads scored in two experiments: wild type, 26; *D mut*, 12. *P=0.05. Student's two-tailed *t*-test conducted on replicate averages; homoscedasticity assumed.

**Fig. 5.**
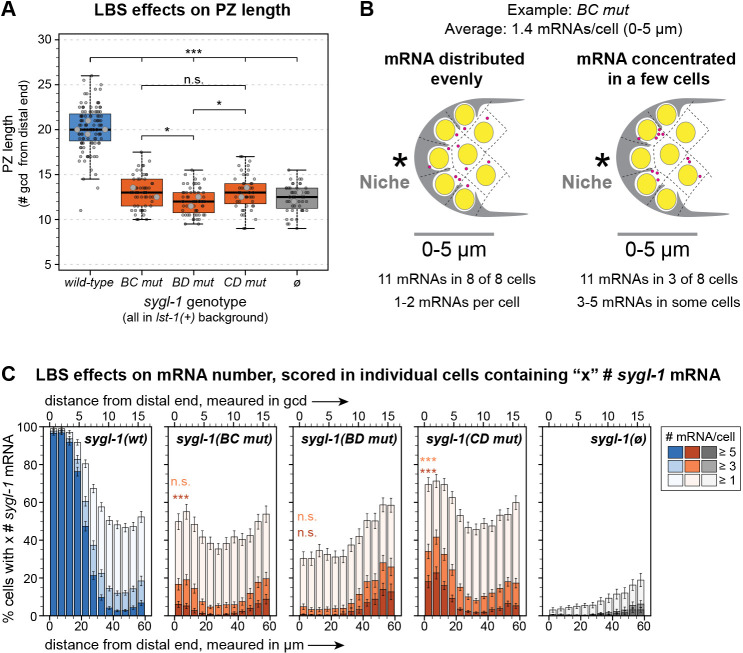
**Solo LBSs are similar but not identical.** (A) Boxplot showing the effect of LBS mutations on PZ length. Boxplot conventions and statistical tests as in Fig. 4B,C. Total gonads scored in two to four experiments: wild type, 111; *BC mut*, 57; *BD mut*, 59; *CD mut*, 48; *sygl-1(ø)*, 48. *P<0.05 (*P*=0.04 in both cases); ***P<0.0001. All other pairwise comparisons are not significant (n.s., *P*>0.05). Not all n.s. comparisons shown. (B) Two explanations of an average of 1.4 mRNA molecules per cell in a troop of eight cells (see Fig. S4A). Left: One or two mRNA molecules present in each cell. An even mRNA distribution might be explained by false positives. Right: No RNA molecules in some cells and three to five in others. Concentrated mRNA is consistent with stochastic transcriptional bursts. (C) Percentage of total nuclei that contain at least one, at least three or at least five *sygl-1* mRNA molecules in each bin of distance from the distal end (Materials and Methods). Position measures as in Fig. 1G. Error bars represent s.e.m. Strains in *lst-1(+)* background. LBS double mutant 0-5 µm bin values were fitted to a linear mixed effects model and Tukey's post-hoc compared LBS double mutant values with the 0-5 µm bin in *sygl-1(ø)*. ***P<0.0001, n.s., *P*>0.01, color-coded for either the ‘at least three’ or ‘at least five’ data.

To assay functions of individual elements in the BCD cluster, we mutated the sequence of each LBS from the canonical 5′-CGTGGGAA-3′ motif to 5′-TGACGTCA-3′ (differences from canonical motif underlined; see Materials and Methods). The three LBSs were mutated singly and in all possible pairs (Fig. 2A). We then subjected all mutants to a series of molecular and biological assays (Figs 2–5). To ensure the LBS changes had not created a novel sequence-specific effect, we made a distinct LBS D mutant, *alt D mut*, which behaved like *D mut* (Fig. S5). All *sygl-1* LBS single and double mutants were homozygous fertile in the presence of wild-type *lst-1.* Molecular assays were therefore performed with *lst-1(+)* to ensure a healthy germline (Fig. 1A,B), but stem cell assays were carried out in an *lst-1(ø)* background. The following sections describe molecular assays first, then biological assays.

### *sygl-1* LBS mutants fire fewer and weaker active transcription sites

We visualized *sygl-1* RNAs *in situ* with high resolution and spatiotemporal precision using smFISH. Fixed gonads were treated with two probe sets that were distinctly labeled to *sygl-1* exons or introns (Fig. 2A). Fig. 2B shows representative smFISH images for LBS single and double mutants plus two controls, wild-type and *sygl-1(ø)* (Fig. 2A; *ø*, open reading frame deletion). The *sygl-1* intron probe detected nascent transcripts in the nucleus as bright spots (Fig. 2B, green, left column), whereas the *sygl-1* exon probe detected both nuclear bright spots and a multitude of dim spots in the cytoplasm (Fig. 2B, middle column, color scaled by intensity). Overlapping exon and intron probe signals in the nucleus identified *sygl-1* ATSs (Fig. 2B, arrowheads), whereas the dim cytoplasmic spots identified *sygl-1* mRNAs (Fig. 2B, arrows) (for additional *sygl-1* ATS and mRNA validation, see Lee et al., 2016).

We quantified the effects of LBS mutations on transcription in three dimensions with a MATLAB code used previously to score wild-type *sygl-1* transcription (Crittenden et al., 2019; Lee et al., 2016). As established in those earlier works, the percentage of cells with any *sygl-1* ATSs provides a measure of *sygl-1* transcriptional probability, and the intensity of each ATS signal provides a measure of firing strength. When LBS mutants were scored individually, each single mutant lowered transcription substantially, and each double mutant nearly abolished it (Fig. S2). Because the three LBS single mutants all had similar effects (Fig. S2B,D,E), as did the three double mutants (Fig. S2C-E), and because all made far fewer ATSs than wild type (Fig. S2B-E), we pooled ATS data into collective ‘LBS single mut’ and ‘LBS double mut’ datasets (Fig. 2C-F) for further analyses.

Transcriptional probability was scored in cells as a function of distance from the distal end of the gonad. For wild type, probability was highest at the distal end adjacent to the niche and lowered progressively with distance from the end (Fig. 2C), as previously reported (Crittenden et al., 2019; Lee et al., 2016). In LBS single mutants, the probability was lower than in wild type, but similarly graded; in LBS double mutants, the probability was near zero (Fig. 2C, Fig. S2A-C). Overall, transcriptional probabilities were dramatically attenuated in both height and spatial extent along the gonadal axis, with LBS double mutants having the most severe effect. As a complementary measure of transcriptional probability, we scored the number of *sygl-1* ATSs in each nucleus. Because *sygl-1* transcriptional probability is stochastic and unrelated to cell cycle stage (Lee et al., 2016), any nucleus in the dividing pool of germ cells might possess zero to four *sygl-1* ATSs, depending on chromosome replication and probability of *sygl-1* transcription. Consistent with our first measure, the percentage of nuclei with more than one ATS was lower in LBS single mutants than in wild type and even lower in LBS double mutants (Fig. 2D, Fig. S2E). Thus, LBS dose regulates the probability of Notch-dependent *sygl-1* transcription.

We next scored ATS signal intensity as a metric of transcriptional firing strength. Intensity values from individual ATSs were captured in the exon channel and normalized using the average raw intensity of cytoplasmic mRNA (see Materials and Methods). When plotted as a function of position along the gonadal axis, mean ATS intensities decreased as germ cells moved proximally through the GSC pool (Fig. 2E). To estimate the average number of nascent transcripts at each ATS, we divided the average normalized ATS intensities by the average normalized single mRNA intensity (see Materials and Methods). By this measure, an ATS in LBS single mutants generated ∼20% fewer nascent transcripts than wild type, on average, and the rare ATS in LBS double mutants made ∼50% fewer transcripts, on average (Fig. 2F). We conclude that *sygl-1* LBS dose regulates both the probability and intensity of *sygl-1* transcription.

### Single *sygl-1* LBS mutations reduce *sygl-1* expression, and double LBS mutations nearly eliminate it

We next investigated how reduced nuclear transcription in LBS mutants affects abundance of *sygl-1* mRNAs and SYGL-1 protein in the distal gonad. The mRNAs were quantified from the same smFISH images used for ATS analyses, in which LBS mutants were not epitope-tagged. Protein was quantified in strains with a V5 epitope tag inserted at the SYGL-1 C terminus of each LBS mutant (Fig. 1E).

To quantify *sygl*-*1* mRNAs, we used MATLAB to detect and count cytoplasmic spots in the exon channel of smFISH images, as described previously (Crittenden et al., 2019; Lee et al., 2016) (see Materials and Methods, Fig. S3A). In LBS single mutants, mRNA abundance was reduced to less than half of that of wild type (Fig. 3A,C), and its spatial extent along the gonadal axis was shorter than that of wild type by ∼5 µm or one or two cell rows (Fig. 3A, vertical lines). In LBS double mutants, Notch-dependent *sygl-1* mRNA was essentially absent: it was just below background in the distal-most germ cells of *BD mut* and just above background in *BC mut* and *CD mut* (Fig. 3E, insets) (see Materials and Methods).

To quantify SYGL-1 protein, we stained each LBS mutant with a V5 antibody (Fig. S3B,C) and scored fluorescence intensities along the gonadal axis using Fiji (see Materials and Methods). In LBS single mutants, the peaks of SYGL-1 gradients were reduced to about 40% of wild type (Fig. 3B,D), and spatial extents were shorter by about 25% of wild type (∼15 µm or three to five cell rows) (Fig. 3B, vertical lines). In LBS double mutants, SYGL-1 protein expression was at or near zero (Fig. 3F). However, like *sygl-1* mRNA, only *BD mut* lacked distal Notch-dependent signal above background (Fig. 3F, horizontal dashed lines). We conclude that for each *sygl-1* LBS mutant, mRNA and protein levels are affected comparably.

### Single *sygl-1* LBS mutants maintain GSCs in the absence of LST-1, but double LBS mutants do not

The molecular quantification described above was carried out in an *lst-1(+)* background to ensure a healthy germline. However, LST-1 masks stem cell defects in *sygl-1* LBS mutants, because of redundancy (Fig. 1B). To score effects of the LBS mutants on stem cell maintenance, we removed LST-1 genetically by introducing *lst-1(ø)* into each mutant. All three LBS single mutants were fertile in an *lst-1(ø)* background and had a germline of normal organization (Table 1, top). By contrast, all three LBS double mutants were sterile with tiny sperm-filled germlines, the Glp phenotype (Table 1, bottom). Therefore, LBS single mutants must generate SYGL-1 at an abundance above the functional threshold required to maintain adult GSCs, but LBS double mutants do not.

**Table 1.**
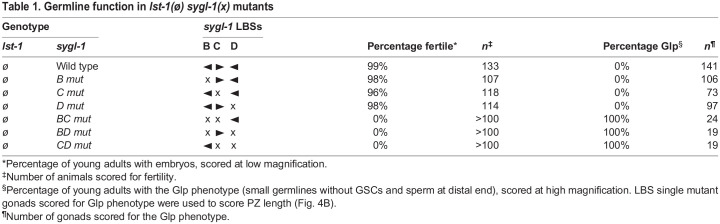
Germline function in *lst-1(ø) sygl-1(x)* mutants

### GSC pool size is reduced in LBS single mutants

Although LBS single mutants maintain adult GSCs, we suspected that their lower SYGL-1 abundance might maintain fewer GSCs and thus shrink GSC pool size. To test this idea, we used three complementary assays (Fig. 4A). All were performed in an *lst-1(ø)* background to eliminate LST-1 redundancy. First, we measured PZ size, a common proxy for GSC pool size, by scoring progenitor cells that had not overtly entered meiotic prophase. Both PZ length along the gonadal axis and total PZ cell number were reduced by about half in all three LBS single mutants compared with wild type (Fig. 4B,C). Second, we measured the distribution of GLD-1, a marker of germ cell differentiation. In *lst-1(ø)* control gonads, GLD-1 abundance increased as germ cells moved proximally through the PZ (Fig. 4D), as previously reported (Brenner and Schedl, 2016; Hansen et al., 2004; Jones et al., 1996). We focused on *D mut* for this and next assay, because the LBS single mutants had behaved similarly overall. In *D mut* gonads, the GLD-1 increase shifted distally compared with the control (Fig. 4D, gray arrow). The likely interpretation of the shorter PZs and distally shifted GLD-1 increase is that germ cells are triggered to begin differentiation more distally in LBS single mutants than in controls. Third, we estimated GSC pool size using the *emb-30* assay to distinguish between germ cells not yet triggered to differentiate and those triggered to differentiate (Fig. 4A, see legend for explanation). This assay provides a rough but more direct measure than other assays. When shifted to restrictive temperature, *emb-30* mutants (Furuta et al., 2000) reveal a distal GSC pool and a proximal pool of GSC daughters starting to differentiate (Cinquin et al., 2010). To estimate GSC pool size in an LBS single mutant, we shifted adults to the restrictive temperature for 12.5 h, stained gonads with a GLD-1 antibody plus a marker of mitosis (PH3 antibody) and counted undifferentiated GSCs in the distal gonad (see Materials and Methods). *D mut* gonads had a visibly smaller GSC pool than the control (Fig. 4E), with 18 cells on average compared with 34 in the control (Fig. 4F). Together, these assays provide complementary lines of evidence that LBS single mutants reduce GSC pool size by about half.

### Individual LBSs have similar but not identical activities

The data presented in Figs 2–4 support the idea that the three LBSs in the *sygl-1* cluster have approximately equivalent roles in regulating *sygl-1* expression and GSC maintenance. Two clues that their roles might not be identical were that *BC mut* and *CD mut* made marginally more *sygl-1* mRNA and protein than did *BD mut* (Fig. 3E,F) and that *D mut* had fewer cells in the PZ than did *B mut* or *C mut* (Fig. 4C). To investigate these differences in more depth, we turned to LBS double mutants. In each double mutant, one LBS is left intact, so the LBS double mutants can thus be used to score activity of that ‘solo’ LBS.

To determine whether the minor molecular differences among LBS double mutants lead to biological differences, we first measured PZ lengths. LBS double mutants do not have a PZ in an *lst-1(ø)* background, so we used an *lst-1(+)* background for this experiment. LBS double mutants had a shorter-than-normal PZ, similar to *sygl-1(ø)* and consistent with their near-complete elimination of SYGL-1 expression (Fig. 5A). However, PZ length was marginally shorter in *BD mut* than in the other LBS double mutants (*P*=0.04), which were not different from each other (*P*=0.10). Because this difference was so slight, we tried another approach. We removed *lst-1* genetically and counted the total number of germ cells in fourth larval stage (L4) larvae. In control *lst-1(ø) sygl-1(ø)* larvae, GSCs begin differentiating in L1 stage (Kershner et al., 2014). Therefore, if L4 larvae have more germ cells than the *lst-1(ø) sygl-1(ø)* control, the solo LBS must have made more SYGL-1 than the null. Germ cells were counted with DAPI and a sperm marker in whole-fixed L4 larvae (see Materials and Methods). *BD mut* made the same number of germ cells as the *lst-1(ø) sygl-1(ø)* control, which does not support GSC self-renewal at any larval stage. *BC mut* and *CD mut*, by contrast, had fivefold more germ cells than the control (Table 2). Therefore, solo LBS C is equivalent to the null, but solo LBS B and solo LBS D must each have weak transcriptional activity, at least in larvae.

**Table 2.**
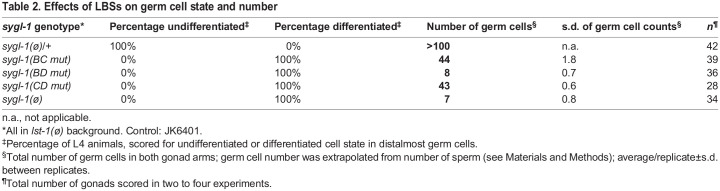
Effects of LBSs on germ cell state and number

The detectable biological activities of *BC mut* and *CD mut* made us wonder whether the mRNA numbers in Fig. 3E, which are averages, might be misleading. Previous work showed that adjacent germ cells can differ dramatically in mRNA number (Lee et al., 2016). Perhaps one or a few cells in LBS double mutants make considerably more mRNA than the average and others make none (Fig. 5B, right). To test this idea, we re-assessed *sygl-1* RNA numbers on a cell-by-cell basis in the distal bin (0-5 µm) of wild type, LBS single mutants, LBS double mutants, and *sygl-1(ø)*. We found that 97% of cells had at least five mRNAs in wild type and at least 86% of cells had at least five mRNAs in LBS single mutants (Fig. S4B). By contrast, *BD mut* and *sygl-1(ø)* had virtually no cells with at least five mRNAs (0.3% and 0%, respectively), but *BC mut* and *CD mut* both had a low percentage of cells with at least five mRNAs (Fig. 5C). This cell-by-cell analysis reveals differences among the LBS double mutants that bulk measurements missed. Those differences are consistent with the conclusion that solo LBS C is less active than solo LBS B or LBS D.

### LBSs function additively and synergistically

Our initial analyses suggested that LBS number plays a crucial role in shaping the *sygl-1* transcriptional gradient: three LBSs generated the normal gradient, two LBSs generated a modest gradient with respect to both abundance and extent along the gonadal axis, and one LBS eliminated the gradient (Figs 2, 3). Fewer LBSs also lowered biological activity compared with wild type. Both PZ size and GSC pool size were roughly halved in LBS single mutants compared with wild type, and both were gone in LBS double mutants (Fig. 4). We next investigated the role of LBS number in heterozygotes (see Materials and Methods). To score molecular and biological outputs from one set of images, we placed all heterozygotes and controls in an *lst-1(ø)* background.

**Fig. 6.**
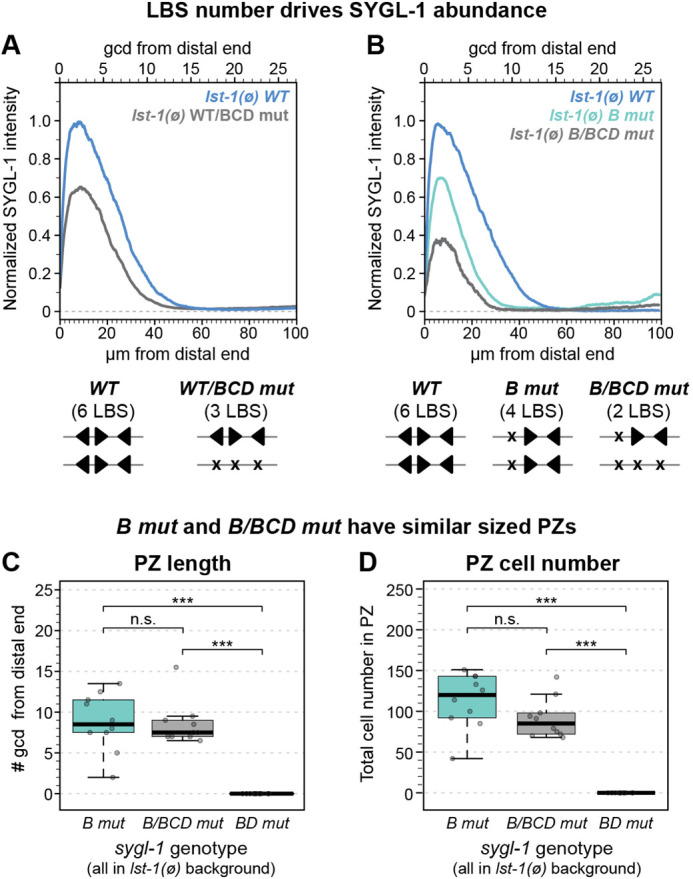
**LBS number is a key factor in determining SYGL-1 abundance.** (A,B) Quantification of V5 immunofluorescence as a measure of SYGL-1 expression. See Materials and Methods for *sygl-1* heterozygote creation. (A) Thirty gonads per genotype scored in two experiments. (B) Total gonads scored in one to four experiments: wild type, 66; *B mut*, 73; *B/BCD mut*, 10. SYGL-1 abundance could not be measured in *lst-1(ø) BD mut* homozygotes, because they lack GSCs and hence lack a germline tissue. (C,D) Analysis of PZ length and PZ cell number in *B/BCD mut* heterozygotes and controls. Boxplot conventions as in Fig. 2F. ***P<0.01, n.s., *P*≥0.01; Student's two-tailed *t*-test (homoscedasticity assumed). Ten gonads per genotype scored from one experiment. (C) Average number of germ cell diameters (gcd) in PZ were manually counted. *P*=0.86 for *B mut* versus *B/BCD mut*. (D) Total number of cells in the PZ counted using Imaris. *P*=0.11 for *B mut* versus *B/BCD mut*.

We first compared SYGL-1 protein abundance in animals homozygous for a wild-type *sygl-1* LBS cluster (*WT*) versus *WT/BCD mut* heterozygotes. We measured protein rather than RNA, because protein is quicker to image and quantify, and because LBS effects on SYGL-1 protein abundance were similar to those on *sygl-1* RNA abundance (Fig. 3). *WT* homozgyotes carry six LBSs, three on each homologous chromosome, whereas *WT/BCD mut* heterozygotes carry only three LBSs, three on the *WT* chromosome and none on the *BCD mut* chromosome. The three LBSs in *WT/BCD mut* produced 65% as much SYGL-1 protein as the six LBSs in *WT*, a bit more than half (Fig. 6A). We next compared protein abundance in strains with six, four and two LBSs: *WT* homozygotes, *B mut* homozygotes and *B/BCD mut* heterozygotes, respectively. We chose the *B mut* chromosome for this heterozygote because all LBS single mutants behaved similarly. The two LBSs in *B/BCD mut* heterozygotes produced about half as much as the four LBSs in *B mut* homozygotes, and about a third as much as the six LBSs in *WT* (Fig. 6B). We were puzzled that SYGL-1 protein in *B mut* was more than half of *WT* when assayed in *lst-1(ø)*, but less than half when assayed in *lst-1(+)* (compare Fig. 6B with Fig. 3B). We found that this difference results from *lst-1* removal, an unexpected result that we have not pursued here (Fig. S7A,B). Regardless, when all were scored in *lst-1(ø)* (Fig. 6B), LBS number determined relative SYGL-1 abundance, consistent with findings when all were scored in *lst-1(+)* (Figs 2, 3). We conclude that LBSs can act additively.

*B/BCD mut* heterozygotes in *lst-1(ø)* possessed a PZ and were fertile (100%, *n*=12). The *B/BCD mut* PZs were remarkably similar to *B mut* PZs in both length and number (Fig. 6C,D). *B/BCD mut* heterozygotes therefore made sufficient SYGL-1 to maintain GSCs into adulthood. These results have two important implications. First, the ability of two LBSs in *cis* to maintain GSCs into adulthood is evidence for LBS synergy. Second, the similarity between *B/BCD mut* and *B mut* PZ sizes is evidence for the gradient acting in a step-wise fashion to regulate both self-renewal and differentiation (see Discussion).

## DISCUSSION

### LBS additivity and synergy control *sygl-1* gradient shape

Three closely spaced Notch-dependent CREs, called LBS B-D, drive *sygl-1* expression in the distal gonad in response to niche signaling. To explore activities of LBSs within this cluster, we generated all possible LBS single, double and triple mutants via CRISPR/Cas9 gene editing of the endogenous *sygl-1* locus. We examined how LBS mutations impacted the gradient shape, which we define according to its peak in the distal end, its extent along the gonadal axis, and the total *sygl-1* present (Table 3). The three LBS single mutants (*B mut*, *C mut* and *D mut*) all reduced *sygl-1* expression by more than half and had similar effects on the shape of the *sygl-1* gradient, particularly the extent of expression along the gonadal axis (Fig. 3A-C). Therefore, three individual LBSs had similar roles within the cluster. A corollary is that the activities of the remaining LBS pairs in each LBS single mutant were also similar despite differences in spacing and polarity (Table 4). Thus, LBS activities within the *sygl-1* cluster can tolerate differences in orientation and spacing, though the limits of that tolerance have not been tested. Each of the three LBS double mutants (*BC mut*, *BD mut* and *CD mut*) reduced *sygl-1* expression to nearly zero, meaning that a cluster requires at least two LBSs to drive a gradient of *sygl-1* expression.

**Table 3.**
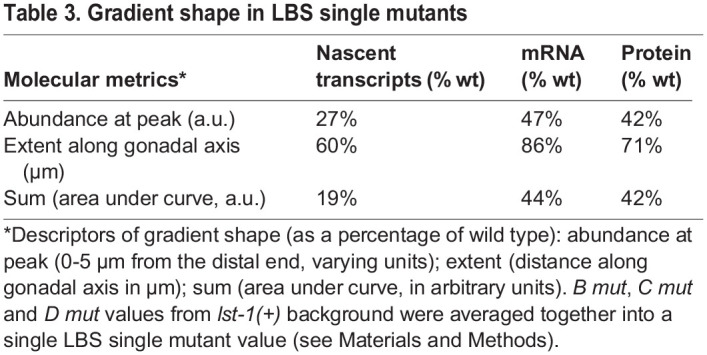
Gradient shape in LBS single mutants

**Table 4.**
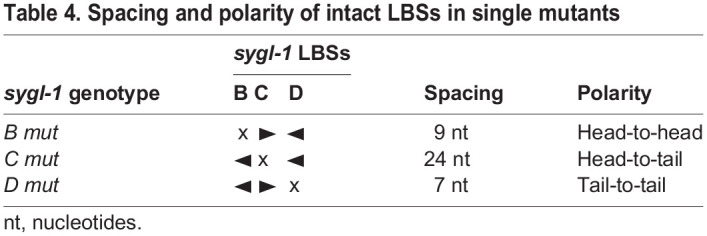
Spacing and polarity of intact LBSs in single mutants

Our *in vivo* approach to functional *cis*-regulatory element analysis enabled us to link LBS function and gradient shape to effects on stem cell maintenance. When tested for biological activity, the *sygl-1* LBS single mutants all maintained GSCs into adulthood in the absence of LST-1, but had lower biological activity than the wild-type LBS cluster (Fig. 4). By contrast, the three LBS double mutants made little or no *sygl-1* RNA and protein and did not maintain GSCs in the absence of LST-1 (Fig. 3E,F, Table 1). Quantitative comparisons of the *sygl-1* gradient and stem cell maintenance in LBS single, LBS double, and heterozygous LBS mutants demonstrate that LBS elements can act both additively and synergistically. In support of LBS additivity, decreases in LBS dose correspondingly shrank the *sygl-1* gradient and reduced PZ size. Evidence for synergy was found in a major exception to this simple rule. The *B/BCD mut* heterozygote, with two LBSs in *cis*, made dramatically more SYGL-1 than LBS double mutants, which possess two LBSs in *trans* and maintained GSCs into adulthood. Traditionally, synergy between *cis*-regulatory elements is deduced when the transcriptional readout from neighboring CREs is greater than the summed readout of separate CREs (Carey, 1991; Ptashne, 1988). By this same logic, we conclude that LBSs in *cis* act synergistically to promote *sygl-1* expression. Synergy between neighboring LBSs also explained another result that was puzzling at first. A solo *sygl-1* LBS drove little or no expression in LBS double mutants and had little or no biological activity, but removal of any one LBS reduced expression substantially (Table 5). For example, the remaining solo LBS C in *BD mut* animals had no detectable activity (Table 2, Fig. 5C), but removal of LBS C in *C mut* animals had a substantial effect (Figs 3, 4, Fig. S4B). How can an individual LBS have no activity in one context and be so important in the other? Synergy between neighboring LBSs within a cluster is the simplest explanation that solves this conundrum and explains why at least two LBSs are required to form a *sygl-1* gradient.

**Table 5.**
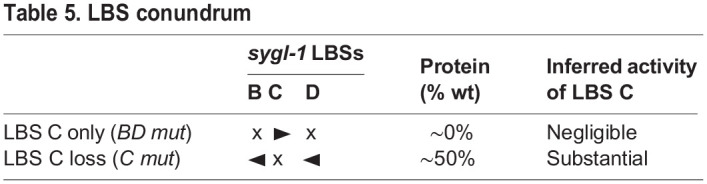
LBS conundrum

Notch-dependent CREs are also synergistic in flies and mammals, but their mechanism is different. In flies and mammals, Notch-dependent CREs in head-to-head polarity orient transcription factor complexes to interact cooperatively at a molecular interface that is not conserved in *C. elegans* (see Introduction). However, LBS polarity is not crucial in *C. elegans* LBS clusters (Neves et al., 2007; this work). *C. elegans* transcription factor complexes may interact via a different interface or *C. elegans* LBS synergy may employ a distinct mechanism. For example, neighboring LBSs may enhance LAG-1/CSL occupancy at the cluster. Like many transcription factors, CSL proteins rapidly bind and release sites with dwell times in the order of one or a few seconds (Falo-Sanjuan et al., 2019; Giaimo et al., 2021; Gomez-Lamarca et al., 2018) and yet their transcriptional bursts last for much longer (10-70 min for *sygl-1*) (Falo-Sanjuan et al., 2019; Lammers et al., 2020; Lee et al., 2019). One can imagine that as LAG-1 releases from an LBS, the existence of a neighboring LBS increases its rebinding. This idea is consistent with the increased occupancy of CSL proteins observed upon Notch activation, especially in genes with clusters of binding sites (Castel et al., 2013; Housden et al., 2013; Wang et al., 2014). Another possibility is ‘assisted loading’, whereby binding of one LAG-1 protein to an LBS indirectly facilitates binding of another. LAG-1 binding, for example, might increase chromatin accessibility and/or concentrate factors that aid LAG-1 recruitment or binding (Ezer et al., 2014; Falo-Sanjuan and Bray, 2020). Both possibilities are consistent with our finding that wild-type clusters with three LBSs drive dramatically more transcription than mutant clusters with two LBSs and again dramatically more transcription than clusters with only a single remaining LBS. Thus, both LBS number and configuration impact cluster activity. We suggest that the combination of LBS additivity and synergy reported here may apply to homotypic CRE clusters more broadly.

### SYGL-1 gradient drives both self-renewal and the transition to differentiation

The SYGL-1 gradient peaks distally in the GSC pool and tapers off more proximally as GSC daughters begin differentiation. An attractive concept is that the extent of SYGL-1 protein along the gonadal axis determines the size of the GSC pool (reviewed by Hubbard and Schedl, 2019). According to this idea, GSCs self-renew when SYGL-1 is present, but differentiate upon SYGL-1 loss. This model was based on the finding that expansion of the SYGL-1 gradient enlarges the GSC pool or can even form a tumor, depending on the extent of the expansion (Shin et al., 2017). However, the effect of a smaller SYGL-1 gradient had not been explored. Here, we report the generation of LBS mutants that shrink the SYGL-1 gradient but do not abolish it. These mutants make enough SYGL-1 to maintain GSCs without its redundant counterpart LST-1, but they also reduce GSC pool size. Therefore, a SYGL-1 increase enlarges the GSC pool (Shin et al., 2017) and a SYGL-1 decrease shrinks it (this work). We can now conclude definitively that the extent of the SYGL-1 gradient determines GSC pool size and specifies the transition from self-renewal to differentiation.

Our measurements of both SYGL-1 gradient parameters and effects on GSC maintenance provide insights into how the gradient is translated into biological activity. Most telling were values in an LBS single mutant and the *B/BCD mut* heterozygote, in which both gradient and biological activity could be measured in an *lst-1(ø)* background (Fig. 6). *B mut* and *B/BCD mut* both made enough SYGL-1 to maintain a fertile germline with PZ and GSCs, but *B mut* made much more SYGL-1 than did *B/BCD mut* (70% versus 40% wild type, respectively, at the peak, and 60% versus 30% wild type summed under the curve) (Fig. 6B). Therefore, a SYGL-1 abundance of ≥40% wild type is above the functional threshold to maintain GSCs. However, a minimal functional threshold could not be defined with our current mutations. It was striking that PZ sizes were similar in *B mut* and *B/BCD mut* despite their distinct SYGL-1 levels. The peak SYGL-1 abundance in *B mut* was nearly twice that of *B/BCD mut*, but the extent of SYGL-1 along the gonadal axis was similar in both. The simplest explanation is that any SYGL-1 abundance above its functional threshold is sufficient to maintain GSCs and that the point along the axis where SYGL-1 drops below that threshold is the site where GSC daughters are launched to begin differentiation. The gradient therefore appears to establish a step function that drives renewal above the threshold and differentiation below the threshold.

### Future directions

This work combines precise genetic manipulation with multiple molecular and biological readouts to analyze the function of *cis*-regulatory elements *in vivo*. Our results provide a new understanding how one cluster of three Notch-dependent elements regulate the *sygl-1* gradient and pattern the stem cell decision between self-renewal and differentiation. However, it also paves the way for many additional analyses, both of this *sygl-1* LBS cluster and others. For this cluster, more in-depth analyses could define the SYGL-1 functional threshold more precisely; show how cluster composition affects the dynamics of transcriptional bursting; and probe how transcriptional regulation can work with regulators of *sygl-1* RNA and protein stability to shape the SYGL-1 gradient and pattern the germline. The analysis of other element clusters will be essential to test the generality of what we learned with the *sygl-1* LBS cluster and its control of the *sygl-1* gradient. The long-term goal must be to untangle the relative contributions of factors that converge on regulation of developmental decisions – autonomous factors within the tissue as well as non-autonomous factors within the organism and from the environment. Such deep analyses will define principles that shape regulatory networks fundamental to all areas of development.

## MATERIALS AND METHODS

### *C. elegans* strains and nomenclature

See Table S1 for a list of genotypes. Throughout, ‘*ø*’ refers to a null mutant deleted for the coding region; ‘*+*’ refers to a wild-type gene. For simplicity, ‘wild-type (wt)’ can refer to either an unmanipulated animal (e.g. Fig. 2) or a modified animal (e.g. V5-tagged *sygl-1* or *lst-1* deleted) with specifics clarified in text, legends, and Table S1. Similarly, LBS mutants are referred to with the same abbreviation (e.g. *D mut*) with specifics of epitope tagging and genetic background clarified in text and legends. Unless otherwise noted, all strains were maintained at 20°C with standard culture methods (Brenner, 1974). The balancer used for *LG I* was *hT2[qIs48]* (Siegfried and Kimble, 2002).

### CRISPR/Cas9 gene editing

No transgenes were used; all genetic edits were carried out by CRISPR/Cas9 genome editing at the endogenous loci. Recombinant Cas9 protein (Paix et al., 2015), single-stranded DNA oligo repair templates complementary to the non-target strand (Paix et al., 2014; Richardson et al., 2016), custom crRNAs (Integrated DNA Technologies), and tracrRNA (Integrated DNA Technologies) were injected into the *C. elegans* germline. A co-conversion approach was used (Arribere et al., 2014): single-stranded DNA repair templates and crRNAs were designed against both the *sygl-1* mutation of interest and a *dpy-10* co-injection marker. Edits of the *dpy-10* co-injection marker create visible phenotypes, decreasing the number of animals that must be screened for the desired *sygl-1* edit by PCR. The *dpy-10* repair and crRNA sequences were from the Andrew Fire Lab (Stanford University, CA, USA) (repair: 5′-CACTTGAACTTCAATACGGCAAGATGAGAATGACTGGAAACCGTACCGCATGCGGTGCCTATGGTAGCGGAGCTTCACATGGCTTCAGACCAACAGCCTAT-3′; crRNA: 5′-GCTACCATAGGCACCACGAG-3′). Final concentrations in the injection mix were as follows: *dpy-10* repair, 1.34 µm; *dpy-10* crRNA, 4 µm; gene-specific repair, 4 µm; gene-specific crRNA, 9.6 µm; tracrRNA, 13.6 µm; recombinant Cas9 protein, 25 µm.

### Scarless CRISPR/Cas9 genome editing

To create a canonical LBS A motif (Fig. S1C,D), we followed a two-step CRISPR/Cas9 editing protocol. The first step creates the point mutation and replaces a stretch of 23 nt in the *sygl-1* 5′ flanking sequence with *dpy-10* protospacer sequence; the second step removes the *dpy-10* sequence to create a scarless edit (El Mouridi et al., 2017). After completing the first step of this protocol, we found that mutating LBS A to a canonical LBS produced SYGL-1 at levels similar to those of wild type and we therefore abandoned the second step.

### Design of LBS mutation

Both the 5′-TGACGTCA-3′ and 5′-GGATCCAA-3′ LBS mutations were designed to mimic LBS mutations from the literature for which loss of function had been demonstrated (Choi et al., 2013; Christensen et al., 1996; Kershner et al., 2014; Neves et al., 2007; Yoo et al., 2004). Mutating the endogenous LBSs presented a genotyping challenge in that the nucleotides in the LBS mutation are the only change from wild type. Thus, we mutated five nucleotides and designed mutant sequences that added a restriction enzyme cleavage site (AatII for 5′-TGACGTCA-3′ and BamHI for 5′-GGATCCAA-3′). Additionally, LBS mutation disrupts the PAM sequence. We alternatively found that designing PCR primers for which the 3′ ends overlapped with the mutated five nucleotides successfully generated either mutant-specific or wild type-specific PCR product.

### *sygl-1* gene diagrams

In Fig. 2A, we show a predicted transcription start site; this transcription start site was drawn using annotation and RNA sequencing data from WormBase version WS280.

Gene diagrams of *sygl-1* are all drawn to scale except that smFISH probes in Fig. 2A are drawn larger than to scale for visibility; the center of each magenta or green line in Fig. 2A indicates the 5′ end of each 20nt-long smFISH probe.

### Immunostaining

#### General staining protocol

Immunostaining was performed as described by the solution extrusion method described by Crittenden et al. (2017) with minor modifications. Specific protocol adaptations are listed in sections below. Unless otherwise noted, animals were dissected at L4+24 h at 20°C in a glass Petri dish with ∼10 ml of 1× PBS + 0.1% Tween-20 (PBSTw) + 0.25 mM levamisole. Samples were moved to a 1.5 ml Eppendorf tube, fixed in paraformaldehyde (Thermo, 28908), washed in 1 ml PBSTw, then permeabilized and washed briefly three times in PBSTw before blocking in PBSTw+0.5% bovine serum albumin (PBSB) for at least 30 min at room temperature. Samples were incubated in the primary antibody solution at 4°C overnight, then washed three or four times in 1 ml PBSTw before adding 100-200 μl of secondary antibody solution. Samples were incubated in the dark at room temperature for 1 h. Another series of three or four PBSTw washes was repeated, shielding samples from light, then samples were mounted in ProLong Gold antifade reagent (Fisher, P36930) on glass slides (FisherFinest Premium, 12-544-1) with 22×22 mm coverslips (Azer Scientific, ES0107052) and cured in a dark drawer at room temperature prior to imaging (cure times overnight to several days).

#### V5 epitope tag staining

For V5 epitope tag staining, samples were fixed in 3% paraformaldehyde for 20 min then permeabilized in 0.5% Triton X-100 in PBS for 5 min. Primary antibody used was mouse anti-V5 antibody (1:1000 in PBSB, SV5-Pk1, Bio-Rad, MCA1360, 1 mg/ml); secondary antibody was donkey anti-mouse Alexa 555 (1:1000 in PBSB, Thermo Fisher Scientific, A31570, lot 1117032) with 0.1 μg/ml DAPI. Antibody solutions were removed with two quick (ten inversions of the tube) and two long (10-15 min on rocker) washes. Samples were mounted in 12 µl ProLong Gold Antifade (Thermo Fisher Scientific, P36930 or P10144). Note that the V5 antibody non-specifically stains the distal tip cell body.

#### GLD-1 and PH3 staining

The same staining protocol was used for GLD-1 staining (Fig. 4D) and the *emb-30* assay (Fig. 4E,F), except that in the *emb-30* assay animals were maintained at different temperatures and staged differently (see ‘*emb-30* assay for number of GSCs’ section below). Note that for simplicity, the GLD-1 staining shown in the schematic in Fig. 4A is a representation of wild-type animals, not the *lst-1(ø)* control animals. This work is consistent with previously published GLD-1 staining in *lst-1* null animals; GLD-1 is brighter in the distal-most region and shifted distally compared with wild type (Brenner and Schedl et al., 2016).

Samples were fixed in 4% paraformaldehyde for 10 min then permeabilized in PBSB with 0.5% Triton X-100 for 10 min. Primary antibodies used were rabbit anti-GLD-1 (1:200 in PBSB, gift from T. Schedl; Jones et al., 1996); mouse anti-phospho-histone H3 (Ser10) (1:200 in PBSB, Cell Signaling Technology, 9706L, lot 10); secondary antibodies were donkey anti-rabbit Alexa 647 (1:1000 in PBSB, Invitrogen Molecular Probes, A31571, lot 1252811), donkey anti-mouse Alexa 555 (1:1000 in PBSB, Thermo Fisher Scientific, A31570, lot 1117032) with 0.1 μg/ml DAPI. Antibody solutions were removed with two quick (ten inversions of the tube) and one long (15 min) wash. Samples were mounted in 18 µl ProLong Glass Antifade Mountant (Fisher, P36984).

#### Immunostaining for PZ analysis

For PZ size counts, a similar protocol to that described above was used except animals were fixed for 15 min in 2% paraformaldehyde, permeabilized for 15 min in 0.5% Triton X-100, blocked for at least 30 min in PBSB, and stained with mouse anti-DAO-5 (1:100 in PBSB) (Developmental Studies Hybridoma Bank; Hadwiger et al., 2010). Some PZ length counts used a shortened method of the above protocol: fixation for 10 min in 4% paraformaldehyde in PBSTw, followed by one quick PBSTw wash, 5 min permeabilization in 0.1% Triton X-100 in PBS, then one wash in PBSTw and mounted in Vectashield plus DAPI (Vector Laboratories, H-1200).

#### Image acquisition

Images were captured on a Leica SP8 confocal microscope. Gonads were imaged from top to bottom with a *z*-slice depth of 0.5 µm. Channels were acquired sequentially (between stacks). V5 signal was excited at 561 nm (0.5%, DPSS, 20 mW) and signal was acquired at 564-615 nm (gain 70) with 16 line averages and two frame accumulations using a 63× objective at 150% zoom and an 8000 Hz scan head; GLD-1 signal was excited at 633 nm (0.2%, HeNe, 2.5 mW) and acquired at 564-614 nm (gain 70, two line averages) using a 40× objective and 125% zoom or a 63× objective and 100% zoom and a 400 Hz scan head; PH3 signal was excited with 561 nm (0.5%, DPSS, 20 mW) and collected at 564-615 nm (gain 80, one line average) using a 63× objective and 100% zoom and a 400 Hz scan head; DAPI was excited at 405 nm (0.8-1.2%, UV, 50 mW) and signal acquired at 412-508 nm (gain 500-700).

### Fluorescence quantification

Fiji (‘Fiji Is Just ImageJ’) was used to quantify fluorescence pixel intensities. Workflow was as described by Haupt et al. (2019) and Brenner and Schedl (2016). A Fiji macro was written to automatically create sum *z* projections and save the output as TIFFs. A 50-pixel wide freehand line was drawn from the distal tip cell down the germline image. Fiji Plot Profile tool was used to copy pixel intensity data and paste into Microsoft Excel.

#### Background subtraction

In Microsoft Excel, an average pixel intensity for each genotype was created by averaging values from individual germlines at each *x* coordinate. Then, the average pixel intensity per genotype was background subtracted using the average pixel intensity from the negative control (no V5 tag); the exception was Fig. 1, for which the untagged JK5622 *sygl-1(ø)* control values were not subtracted in order to display them in comparison to *BCD mut* values. For all other experiments, the untagged control was N2 (wild type).

#### Normalization of background-subtracted pixel intensities

After pixel intensities were processed as above, intensities were normalized in one of two ways. For Figs 1, 3 and Fig. S7, the values at each *x* coordinate were divided by the maximum intensity from the positive control to transform intensities into a percentage of control. The positive control for Figs 1 and 3 is JK6002 *sygl-1::V5(wt)* and for Fig. S7 is JK6431 *lst-1(ø) sygl-1::V5(wt)* homozygotes.

For the GLD-1 data in Fig. 4D, processed pixel intensities were normalized against an internal control: JK4864 (Table S1) worms expressing a GFP reporter in somatic cells were grown on the same plates and dissected in the same dish as the test worms. The JK4864 control worms have normal GLD-1 expression and the peak value in the JK4864 data was set to 1.0 for each slide.

#### s.e.m. shading on plots

s.e.m. values were calculated on background-subtracted pixel intensity values; values from different experiments were averaged together and then normalized. Standard error was calculated using the number of experiments as *n*. Thus, the s.e.m. shading reflects the spread of pixel intensity values on different slides imaged on different days.

s.e.m. shading was used on immunostaining data for some figures but not others. For SYGL-1 abundance figures lacking s.e.m. shading, we normalized background-subtracted values to their respective controls (stained in parallel) before averaging together values from different replicates, which ensures that mean values are robust against day-to-day variation in pixel intensity.

#### Total protein (area under the curve)

To calculate total protein quantification (Fig. 3D), arrays *x* (distance values in µm) and *y* (normalized SYGL-1 intensity values) were imported into MATLAB version R2015a and the command trapz(X,Y) was used to calculate the area under the curve.

#### Measurements along the gonadal axis

Microns were converted to germ cell diameters (gcd) using a conversion factor of 3.7 μm/gcd, which was calculated by manually measuring cell diameters in Fiji to determine an average distance between the centers of neighboring nuclei (ten cells were measured for one randomly chosen image from each experiment, using both protein staining and smFISH experiments).

### smFISH

The same *sygl-*1 exon and intron probes were used as described by Lee et al. (2016). See also a published smFISH protocol (Lee et al., 2017). Briefly, mid-L4 stage animals were grown on OP50 at 20°C for 24 h, then dissected as described in the ‘Immunostaining’ section above. Animals were fixed in 3.7% formaldehyde (37% formaldehyde Amresco, 0493-500ML) for 15-25 min and permeabilized in RNAse-free PBS (Fisher, BP24384) with 0.1% Triton X-100 for 10-12 min. After an overnight incubation in 70% ethanol [diluted with DEP-C-treated H_2_O (Ambion AM9922)], samples were equilibrated in smFISH wash buffer (Lee et al., 2017) for 15-20 min, then incubated in hybridization buffer (Lee et al., 2017) plus smFISH probe at 37°C for 46-48 h. Lyophilized smFISH probes were resuspended in RNAse-free TE buffer (10 mM Tris-HCl, 1 mM EDTA, pH 8.0) to make a 250 μM stock solution. The *sygl-1* exon-specific probe set includes 31 unique oligonucleotides labeled with CAL Fluor Red 610 and was used at a final concentration of 0.25 μM. The *sygl-1* intron-specific probe set includes 48 unique oligonucleotides tagged with Quasar 570 and was used at a final concentration of 0.50 μM. *sygl-1* smFISH probe sequences are included in Table S2. Samples were hybridized at 37°C overnight, then washed with smFISH wash buffer and 1 μg/μl DAPI at 37°C for 40-50 min. Finally, samples were resuspended in 12 μl Prolong Gold antifade reagent (Fisher, P36930), mounted on glass slides, and cured unsealed in a dark drawer for at least 24 h to several days before imaging.

#### Image acquisition settings

Images were captured on a Leica SP8 confocal microscope with the same hardware and Leica software as described by Lee et al. (2016). Gonads were imaged from top to bottom with a *z*-slice depth of 0.3 µm, with a 63× objective at 300% zoom. Channels were acquired sequentially in the following order: *sygl-1* intron Q570 probe was excited at 561 nm (3%, DPSS, 20 mW) and signal was acquired at 564-588 nm (gain 40); *sygl-1* exon C610 probe was excited at 594 nm (3%, HeNe, 2.5 mW) and signal acquired at 600-680 nm (gain 40); DAPI was excited at 405 nm (0.8-1.2%, UV, 50 mW) and signal acquired at 412-508 nm (gain 500-700). Six of nine experiments were imaged between frames with a 400 Hz scan head and line average of six for RNA channels or three for DAPI; three of nine experiments were imaged between stacks with an 8000 Hz scan head and line average of 16 for introns, 32 for exons, and eight for DAPI with two frame accumulations on each channel.

#### Representative images

Image contrast was adjusted in Fiji equivalently for all main images and for all insets. Contrasts for intron channels differ between main images and insets to highlight ATS number in main images and ATS intensity in insets. Exon channels were pseudocolored with Fiji default Fire LUT: this distinguishes cytoplasmic mRNA as a dimmer population than the ATS without saturating ATS. All gonad images were taken from the same experiment.

### MATLAB analysis of smFISH data

#### Removal of data points

Some images in a few experiments displayed bright, nonspecific signal outside the gonad tissue that interfered with detection. Images for which the nonspecific signal was greater than 8 μm^2^ were removed [one *wt*, ten *C mut*, six *D mut*, three *CD mut*, one *sygl-1(ø)*]. Additionally, a small number of images generated errors in MATLAB related to reading the image files (e.g. nuclei imaged too close to edge of frame) and were also removed [five *C mut*, five *BD mut*, three *CD mut*, one *sygl-1(ø)*]. The total numbers of images quantified are reported in Fig. S2E.

#### MATLAB code

The MATLAB code analysis has been previously described (Lee et al., 2016, 2022; Crittenden et al., 2019). Code modifications described by Crittenden et al. (2019) were also applied to this work. Threshold values were set as 1.0 thresForIntron and 0.55 thresForExon for all images. Nuclear detection settings are as follows: thresForNuc, 0.5; radius, 2.5; nrange 1.2-2.8; sensi, 0.96. To define cell boundaries for number of mRNA molecules per cell calculations, a Voronoi with 3 µm limit was used [as used by Crittenden et al. (2019) and Lee et al. (2016); does not include center of rachis].

#### Cytoplasmic spots versus mRNA

The dim cytoplasmic spots ranged slightly in intensity and size. For wild type and each LBS mutant, ∼60% of cytoplasmic spots had a dim intensity distribution with a low coefficient of variation, consistent with a single mRNA (Fig. S3A) (see also Lee et al., 2016). Approximately one-third of the cytoplasmic spots in each genotype were slightly larger and their intensity values were approximately twice that of the single mRNA population, consistent with two mRNA molecules. Rare spots had three or more mRNA molecules per cytoplasmic spot. Where possible, the number of mRNA molecules were reported rather than the number of cytoplasmic spots, but the #mRNA/cell values represented in Figs 3A,E, 5C and S4B are the number of cytoplasmic spots.

#### Background level and extent of *sygl-1* mRNA gradient

The background level in Fig. 3A,E is 0.956 cytoplasmic spots (or ∼1.25 mRNA molecules; see Fig. S3A). This background was determined by calculating one standard deviation above the mean number of cytoplasmic spots detected in the 45-50 µm region of *sygl-1(ø)* control gonads. Local minimums indicate the end of the Notch-dependent *sygl-1* gradient because the slight increase in RNA in the 40-60 µm region is consistent with the rise in proximal Notch-independent *sygl-1* expression (see Introduction).

#### Normalization of ATS pixel intensities

To control for image-to-image intensity changes, raw exon channel ATS values from each gonad were divided by the mean intensity for the raw single mRNA population (the population of cytoplasmic spots estimated to be one mRNA) within the same gonad. Then, the mean of all the single mRNAs was transformed to ten. Thus, a normalized ATS intensity of 50 was five times brighter than the mRNA within the same image. Normalized ATS intensities were divided by ten to estimate the number of nascent transcripts per ATS (Fig. 2F); this estimate is rough because ATS include transcripts of varying lengths that bind varying numbers of probes.

#### Number of ATS per nucleus pie charts (Fig. 2D)

The percentages of nuclei containing *x* number of ATS were calculated individually for each experiment. The percentages in the pie charts are the average percentage of all the experiments in the datasets. For example, the LBS double mutant dataset contained 94 nuclei and only one of those nuclei contained three ATSs. That 3-ATS nucleus came from one experiment with 21 total nuclei; that experiment's 3-ATS percentage was 4.8%. The other seven experiments that contained any ATS-positive nuclei had 0% nuclei with three ATSs and thus the overall average was 0.6%.

#### Summary of *sygl-1* mRNA data (Fig. 3D)

The total number of single mRNA molecules per experiment was divided by the number of gonads in that experiment to calculate ‘Ave # mRNA per gonad’; standard deviation was calculated between experimental averages. The peak number of mRNA molecules was the maximum number of cytoplasmic spots detected (bar height in 0-5 µm bin of average #mRNA/cell plots) as a ratio to wild type; standard deviations were calculated between the average values for each independent experiment. The ‘Total mRNA’ data are the ‘Ave # mRNA per gonad’ data represented as a ratio to wild type.

### Germline function

#### Fertile adults

To score the percentage of fertile animals, F1 homozygous L4 s were picked from balanced *lst-1(ø) sygl-1(LBS single mut)* stocks onto a fresh plate; 24 and 48 h later, one L4+24 or L4+48 adult was singled onto a plate to lay embryos for 1.5-3.5 h. The percentage fertility of F2 homozygotes was scored (gravid or not fertile) at low magnification on a Zeiss Discovery.V12 microscope 4 days after eggs were laid. Progeny from a total of eight different adults per genotype were scored. LBS double mutant animals were maintained for many generations and a gravid homozygous animal was never seen. Additionally, *lst-1(ø) sygl-1(LBS double mut)* homozygous L4s were raised at 15°C, 20°C and 25°C and failed to produce embryos at all temperatures.

#### Glp phenotype

To score the percentage Glp phenotype (germline proliferation abnormal), L4+24 h LBS double mutant adults were assessed at high magnification on a Zeiss Axio Imager.D1 microscope by differential interference contrast. Sperm were observed at the distal tip of all LBS double mutant germlines at 20°C. LBS single mutants were scored as 0% Glp because 100% of germlines had a PZ.

### PZ size

PZ size was assessed in animals grown at 20°C for 24 h past mid-L4. Gonads were extruded, fixed and stained with DAPI or DAPI and DAO-5 antibodies (see ‘Immunostaining’ section above).

#### PZ length

PZ lengths were determined as follows: first, the distal tip cell (DTC) was located, then the number of cell rows between the DTC and the first crescent were counted. Both sides of the germline were counted and averaged together for one PZ length per germline. Note that because a curved metaphase plate can look similar to a crescent cell, the definition of ‘first crescent’ cell included a requirement for multiple nearby crescent cells. For more information on identifying and scoring PZ, see Crittenden et al. (2017). Gonads were visualized on a computer monitor for ease of manual counting; μManager software (a plugin for ImageJ) was used to connect the camera and computer (Edelstein et al., 2010).

#### Number of cells in PZ

A protocol using Imaris software was modified from previous studies (Braude et al., 2015; Gopal et al., 2017; Sadeghi et al., 2014). A surface was drawn to mask the PZ region using the drawing contour mode with 1.7 µm vertex spacing. The PZ boundary along the gonadal axis was chosen by eye on a middle slice where the first crescent cells could be seen, then the outline was copy/pasted to the first and last slices to create a 3D surface. The surface was used to mask the DAO-5 channel. A spots function with an estimated *xy* diameter of 2.0 µm was created in the masked DAO-5 channel. The number of spots was filtered with a quality filter and the threshold on the quality filter was set by moving the slider until no spots were detected outside the germline. Detection was briefly visually checked and the number of spots detected was recorded as the number of cells in the PZ. We also used Imaris to count the number of cells in the PZ for some experiments that were DAPI stained but not DAO-5 stained (Fig. S5E,F, Fig. 6D). The protocol was the same except we used a 2.7 µm estimated *xy* diameter on a masked DAPI channel and spent more time per gonad checking and editing detected spots.

Imaris version 9.3.1 was used on a Dell Precision 5820 with 64-bit Windows 10 Education operating system, an Intel^®^ Xeon^®^ W-1245 CPU @3.70 GHz processor, and 128 GB of RAM. Leica .lif images were converted to .ims images using Imaris FileConverter v9.5.0.

### Sperm counts

Mid-L4 larvae were harvested and stained as whole mounts with the reduction/oxidation method (Finney and Ruvkun, 1990). Samples were fixed in Ruvkun fixation buffer with 1% paraformaldehyde for 30 min, followed by disulfide linkage reduction and incubation in blocking solution (1× PBS with 1% bovine serum albumin, 0.5% Triton X-100, 1 mM EDTA) for 40 min. Samples were then incubated overnight at 4°C with rabbit anti-SP56 (1:200; Ward et al., 1986) in blocking solution. Secondary antibody Alexa 555 goat anti-rabbit (1:1000, Invitrogen Molecular Probes, A21429, lot 1246456) was diluted in blocking solution and incubated with samples for at least 2 h. Samples were washed with blocking solution and 1 ng/µl DAPI for 15 min, then mounted in Vectashield (Vector Laboratories, H1000) for visualization with a Zeiss Axio Imager.D1 fluorescence compound microscope.

#### Calculation of germ cell number

Germlines lacking both *lst-1* and *sygl-1* begin precociously differentiating in L1, two full larval stages earlier than L4, and have completed both spermatogenic meiotic divisions by L3, which creates 16-32 sperm from a total of four to eight germ cells (Kershner et al., 2014). The distal-most cells were scored as undifferentiated or as differentiated. Undifferentiated cells were scored by DAPI morphology. Differentiated cells included meiotic cells, spermatocytes, or fully developed sperm and were scored by SP56^+^ staining and/or DAPI morphology). Estimates of the number of germ cells were extrapolated as follows: one-quarter GSC per sperm, half a GSC per secondary spermatocyte, and one GSC per primary spermatocyte or meiotic cell. Sperm and spermatocytes were counted in one gonad arm in JK5911 and JK6180 and multiplied by two. Sperm were counted in both gonad arms in JK6165 and JK6401.

### Creating *sygl-1* heterozygotes

Heterozygous LBS effects were scored in cross progeny from genetic crosses. All strains contained *lst-1(ø)* for two reasons. First, *lst-1(ø)* allowed us to score GSC maintenance and SYGL-1 abundance in the same animals. Second, *lst-1(ø)* allowed us to distinguish self and cross progeny (see below). Parent strains (see Table S1) for each figure were as follows: Fig. 6A,B: *WT*: JK6431 males×JK6431 hermaphrodites; Fig. 6A, Fig. S6B: *WT/BCD mut*: JK6431 males×JK6600 hermaphrodites; Fig. 6B: *B mut*: JK6517 males×JK6517 hermaphrodites, *B/BCD mut*: JK6517 males×JK6600 hermaphrodites; Fig. S6B, *WT/ø*: JK6431 males×JK6401 hermaphrodites; Fig. S6C: *B mut/BCD mut*: JK6517 males×JK6600 hermaphrodites; *B mut/ø*: JK6517 males×JK6401 hermaphrodites.

To distinguish self- and cross-progeny from each cross, the hermaphrodite parent carried *lst-1(ø) sygl-1(null)* [*sygl-1* null meaning open reading frame deletion (*ø*) or *BCD mut*] heterozygous with a balancer chromosome that drives GFP expression. This meant that homozygous (non-green) self-fertilized progeny from the hermaphrodite parent were 100% sterile and therefore any non-green progeny that maintained GSCs as adults must be heterozygous cross-progeny. To validate this cross method and rule out the possibility that some heterozygous cross progeny are sterile, we first designed a cross with an *lst-1(ø) sygl-1(null)* hermaphrodite that also carried a recessive *rol-6(e187)* allele. In three repeats of this cross, all non-green self (Roller) progeny were sterile and all non-green heterozygous (non-Roller) progeny were fertile and maintained GSCs. The strains reported in Fig. 6 did not include the *rol-6* allele and were scored with the knowledge that all sterile Glp animals were self-progeny.

### *emb-30* assay for number of GSCs

The *emb-30* assay was used to estimate the naïve GSC pool size, as previously described (Cinquin et al., 2010). Temperature-sensitive *emb-30* mutants (Furuta et al., 2000) were grown at permissive temperature (15°C) until 36 h past L4, then shifted to restrictive temperature (25°C) for 12.5 h. Temperature shifts were carried out in a programmable incubator (Echotherm IN35, Torrey Pines Scientific). Gonads were dissected, fixed and stained for GLD-1, PH3 and DAPI (see ‘Immunostaining’ section above). The number of naïve GSCs was estimated by using the multi-point tool in Fiji (Schindelin et al., 2012) to count number of nuclei distal to the GLD-1 staining boundary that were positive for PH3 and/or mitotic by DAPI.

No animals in *lst-1(ø) sygl-1::V5(wt); emb-30* controls (permissive and restrictive) were meiotic, but six of 17 *lst-1(ø) sygl-1::V5(D mut); emb-30* controls were meiotic at permissive temperature. We interpreted this as a consequence of the *emb-30* mutant background and discarded meiotic gonads (5/21 for *D mut*, 0/28 for *wt*) from analysis. As reported previously (Crittenden et al., 2019), we also excluded gonads in which most germ cell nuclei were fragmented (4/21 for D mut, 2/28 for WT), which could not be reliably scored.

### Sources for percentages in Table 3

In Table 3, there are single values that each summarize the ‘abundance’, ‘extent’ and ‘sum’ of nascent (1°) transcripts, mRNA or protein for all three LBS single mutants. Sources for the data that determined each of these numbers are recorded here. Nascent transcript values are as follows: abundance at peak values in Fig. 2C (LBS mut: 14.2±5%; wild type: 51.8±8%); extent values from vertical dashed lines in Fig. 2C (LBS mut: 15 µm; wild type: 25 µm); sum values are an estimate of the total number of primary transcripts per gonad using numbers from Fig. S2E (LBS mut: 5.8±4.1 ATS/gonad * 4.5±0.6 tx/ATS=17 tx/gonad; wild type: 24±8.5 ATS/gonad * 5.6±0.5 tx/ATS=134 tx/gonad). The mRNA values are: abundance from Fig. 3A,C (LBS mut: 29±3 mRNA at peak; wild type: 62±5 mRNA at peak); extent from vertical dashed lines in Fig. 3A (LBS mut: 30 µm; wild type: 35 µm); sum from Fig. 3C (LBS mut: 2616±864 mRNA; wild type: 6274±1478 mRNA). The protein values are: abundance from Fig. 3D (LBS mut: 42±13% of wild type peak); extent from vertical dashed lines in Fig. 3B (LBS mut: 43 µm; wild type: 52 µm); sum from Fig. 3D (LBS mut: 11,557±3678 a.u.; wild type: 2781±5739).

### Statistics

#### Box plots

Box plots were generated with the BoxPlotR web tool (Spitzer et al., 2014). BoxPlotR conventions: box limit: 25th and 75th percentiles; whiskers extend 1.5 times the interquartile range from the 25th and 75th percentiles.

#### Statistical tests

Statistical tests were performed with consultation from the College of Agriculture and Life Sciences Statistical Consulting Lab and tests for normality and variance were standard. Sample sizes are recorded in each legend and are appropriately sized for the conclusion the data support. Student's *t*-test (T.TEST function in Excel) was used whenever two samples could be compared. For multiple pairwise comparisons, we chose a linear mixed effects model. Observations were fitted to a linear mixed effects model (lmer) using lme4 package. R version 4.0.5 (2021-03-31) – ‘Shake and Throw’ and RStudio version 1.4.1106 ‘Tiger Daylily’ were used with Windows 10. The genotype was the fixed effect and the experiment block was the random effect. The emmeans package was used to make pairwise comparisons between genotypes for a given ‘distance from the distal end’ bin.

#### Standard error bars

We used the number of gonads to calculate s.e.m. bars for *sygl-1* mRNA data because there are multiple mRNA/cell measurements from a single gonad. In all other cases, we used the number of experiments to calculate s.e.m. bars because there was only one measurement per gonad.

## Supplementary Material

Supplementary information
