# Supplementary material for: Notch-dependent DNA cis-regulatory elements and their dose-dependent control of C. elegans stem cell self-renewal
Source: Development. 2022 Apr 8;149(7):dev200332. doi: 10.1242/dev.200332 (PMC9058496; doi:10.1242/dev.200332)
Supplement: Supplementary information [file develop-149-200332-s1.pdf]

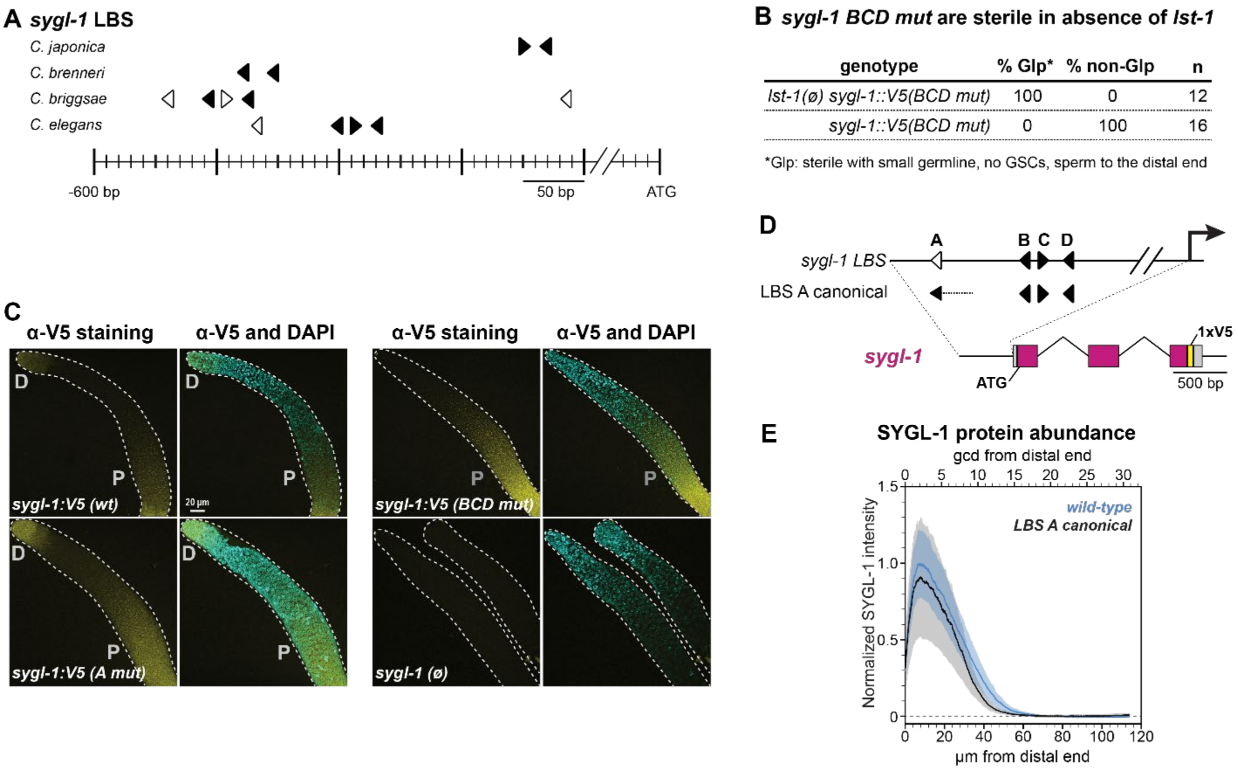

Fig. S1. Additional analyses of *sygl-1* LBS and their mutants

A. LBS clusters in *sygl-1* orthologs. All canonical LBSs (filled black arrows) have the same sequence: 5′ CGTGGGAA 3′; non-canonical sequences (open arrowheads) in *C. briggsae*: 5′ TATGGGAA 3′, 5′ CATGGGAA 3′, and 5′ TGTGTGAA 3′. Spacing between LBSs is as follows: *C. japonica*: 10 bp; *C. brenneri*: 24 bp *C. briggsae*: from upstream to downstream 24 bp, 7 bp, 10 bp, and 252 bp; *C. elegans*: 57 bp, 7 bp, and 9 bp. **B.** Percent of Glp L4 animals was scored. Germlines were assessed on DIC at high magnification. **C.** Notch-independent SYGL-1 expression in the proximal gonad is present in LBS mutants. Representative images of dissected gonads show both distal (D) and proximal (P) SYGL-1 expression. Note: *sygl-1* expression becomes brighter nearer the oocytes in the proximal germline (Lee et al., 2016); germlines lacking SYGL-1 are smaller in size and thus at this magnification more of the brighter proximal expression can be seen in germlines lacking SYGL-1. Strains and conventions are as in Fig. 1F. **D-E.** Expression of SYGL-1 protein in mutants with canonical LBS A sequence. **D.** Schematic of LBS A canonical mutation. *sygl-1* LBS A sequence was mutated from 5′ AGTGGGAA 3′ to 5′ CGTGGGAA 3′; this mutation also replaced 23 nt downstream of LBS A (5′ AAAAGGACTACTGTAGTCAATAC 3′) with a PAM and *dpy-10* crRNA protospacer (5′ CCGCTCGTGGTGCCTATGGTAGC 3′) (dotted line). Arrowhead and gene diagram conventions match Fig. 1E. **E**. The *LBS A canonical* mutant does not have a major effect on SYGL-1 expression. Quantification of α-V5 immunofluorescence (see Methods). Strain genotypes in Table S1. Total gonads scored in 3 independent experiments: *wild-type(wt)*: 71; *LBS A canonical*: 63; untagged control: 43.

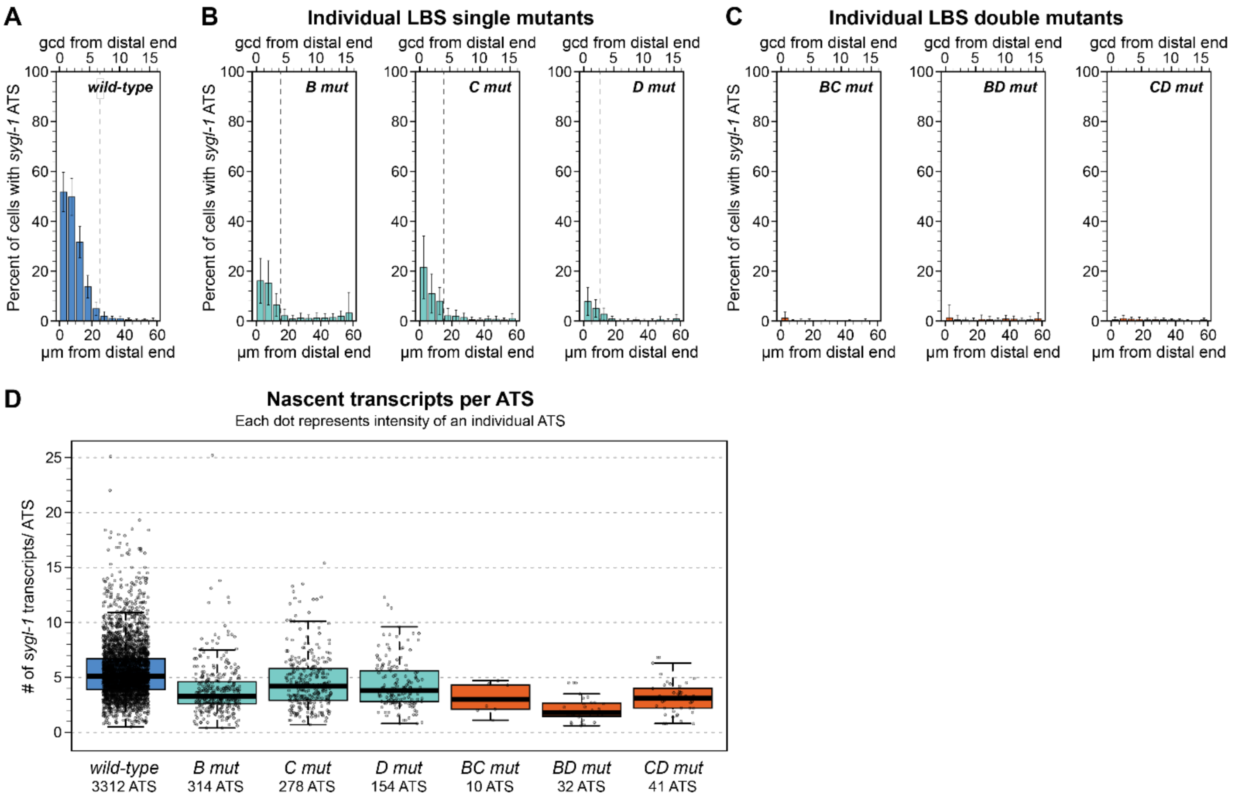

E

Genotype	n	Ave # ATS/gonad	Ave # transcripts/ATS	#ATS per nucleus (%) 1	2	3	4
sygl-1(wild-type)	**140**	**24.0** ± 8.5	**5.6** ± 0.5	**78** ± 8.6	**21.0** ± 7.2	**1.4** ± 1.5	**0.3** ± 0.4
sygl-1(B mut)	**50**	**6.8** ± 3.9	**3.9** ± 0.3	**88** ± 9.5	**10.0** ± 7.7	**0.8** ± 1.2	**0.4** ± 0.6
sygl-1(C mut)	**44**	**7.4** ± 4.6	**4.9** ± 0.7	**83** ± 8.8	**14.0** ± 6.4	**2.8** ± 2.4	**0.3** ± 0.5
sygl-1(D mut)	**65**	**3.1** ± 2.7	**4.7** ± 0.9	**89** ± 8.0	**9.0** ± 6.1	**0.3** ± 0.6	**1.8** ± 2.1
sygl-1(BC mut)	**47**	**0.3** ± 0.4	**3.0** ± 0.2	**100** ± 0.0	**0.0** ± 0.0	**0.0** ± 0.0	**0.0** ± 0.0
sygl-1(BD mut)	**47**	**0.9** ± 1.5	**2.7** ± 1.1	**92** ± 9.3	**5.2** ± 6.0	**1.2** ± 2.4	**1.2** ± 2.4
sygl-1(CD mut)	**76**	**0.7** ± 0.7	**2.6** ± 0.9	**96** ± 5.6	**3.9** ± 5.6	**0.0** ± 0.0	**0.0** ± 0.0
sygl-1(ø)	**51**	**0.0** ± 0.1	**2.2** ± 0.0	**100** ± 0.0	**0.0** ± 0.0	**0.0** ± 0.0	**0.0** ± 0.0

Fig. S2. Transcriptional activation of individual LBS mutants is essentially equivalent

A-C. ATS probability: percentage of nuclei with ≥1 *sygl-1* ATS as a function of distance from the distal end. Conventions as in Fig. 2C. n's listed in Fig. S2E. **A.** Wild-type ATS probability. Data is the same as in Fig. 2C. **B.** LBS single mutant ATS probability. **C.** LBS double mutant ATS probability. **D.** Estimated number of nascent transcripts per ATS for individual LBS mutants. Boxplot conventions as in Fig. 2F; center line: median (*wild-type*: 5.1; *B mut*: 3.3; *C mut*: 4.2; *D mut*: 3.8; *BC mut*: 3.0; *BD mut*: 1.8; *CD mut*: 3.1). **E.** Summary statistics for individual genotypes that were pooled in Fig. 2; see Table S1 for strain genotypes. All *sygl-1* smFISH data is from L4+24 adults. Numbers are mean from each independent experiment plus or minus standard deviation between experimental means. n: number of gonads scored. 9 experiments were performed; data for each genotype comes from at least 3 experiments (# experiments = *wild-type*: 9; *B mut*: 3; *C mut*: 3; *D mut*: 4; *BC mut*: 3; *BD mut*: 3; *CD mut*: 4; *sygl-1(ø)*: 3). Ave # ATS/gonad, total number of ATS divided by the number of gonads. Average # transcripts/ATS, mean normalized ATS intensity divided by 10 to estimate the number of nascent transcripts per ATS, as in Fig. 2F, S2D. #ATS per nucleus, percent of ATS-expressing nuclei that contain the given number of ATS, as in Fig. 2D.

Development • Supplementary information

A

LBS single mutants

Genotype	n	Percentage cytoplasmic spots with x number of mRNAs			
		**x = 1**	**x = 2**	**x = 3**	**x = 4+**
sygl-1(wild-type)	**140**	**61** ± 4	**30** ± 4	**6.2** ± 0.7	**2.3** ± 0.3
sygl-1(B mut)	**50**	**62** ± 4	**33** ± 4	**4.6** ± 0.3	**1.0** ± 0.4
sygl-1(C mut)	**44**	**61** ± 4	**32** ± 5	**5.2** ± 0.5	**1.5** ± 0.2
sygl-1(D mut)	**65**	**59** ± 2	**35** ± 1	**5.0** ± 0.8	**1.1** ± 0.4
sygl-1(ø)	**51**	**73** ± 4	**20** ± 5	**5.8** ± 0.7	**2.0** ± 0.4

n: number of gonads scored
x: number of mRNAs estimated per cytoplasmic spot

LBS double mutants

Genotype	n	Percentage cytoplasmic spots with x number of mRNAs			
		**x = 1**	**x = 2**	**x = 3**	**x = 4+**
sygl-1(wild-type)	**140**	**61** ± 4	**30** ± 4	**6.2** ± 0.7	**2.3** ± 0.3
sygl-1(BC mut)	**47**	**59** ± 7	**37** ± 8	**4.6** ± 1.9	**0.3** ± 0.5
sygl-1(BD mut)	**47**	**60** ± 8	**37** ± 9	**2.9** ± 0.9	**0.6** ± 0.5
sygl-1(CD mut)	**76**	**57** ± 8	**39** ± 9	**3.1** ± 1.3	**0.6** ± 0.4
sygl-1(ø)	**51**	**73** ± 4	**20** ± 5	**5.8** ± 0.7	**2.0** ± 0.4

n: number of gonads scored
x: number of mRNAs estimated per cytoplasmic spot

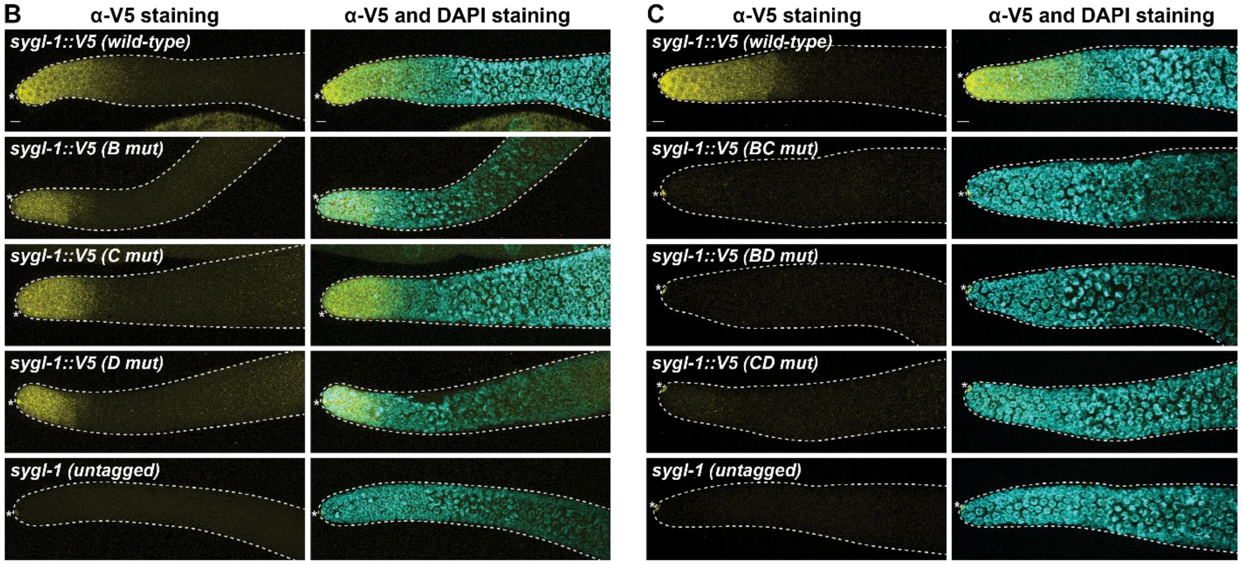

Fig. S3. Characterization of LBS single mutant mRNA and protein data

A. Percent of cytoplasmic spots containing 1, 2, 3, or 4+ mRNA as a percent of the total number of cytoplasmic spots detected. Numbers are averages per replicate plus or minus the standard deviation between replicates. n: number of gonads analyzed, from 3-9 experiments. **B.** Representative images of SYGL-1 protein in LBS single mut gonads. Images were selected from the same experiment, were adjusted with the same contrast values in FIJI, and are maximum z projections. Dashed gray line outlines the gonad and asterisk marks the niche cell body. V5 signal stains the niche cell body nonspecifically. Scale bar: 5 µm. **C.** Representative images of SYGL-1 protein in LBS double mut gonads; conventions as in Fig. S3B.

A Average number of MATLAB-detected nuclei per bin

	Bin number											
**Genotype**	**1**	**2**	**3**	**4**	**5**	**6**	**7**	**8**	**9**	**10**	**11**	**12**
sygl-1(wild-type)	8.5	12.5	15.3	17.3	19.3	20.0	21.5	22.1	22.3	22.1	20.3	9.1
sygl-1(B mut)	7.9	11.9	13.9	15.6	16.4	16.6	17.3	18.3	17.6	17.3	16.5	8.9
sygl-1(C mut)	7.8	12.0	14.3	16.5	16.6	18.0	18.1	19.0	19.5	18.7	19.1	9.0
sygl-1(D mut)	7.8	11.9	14.6	17.5	18.9	20.1	20.4	21.3	20.1	20.0	18.1	8.9
sygl-1(BC mut)	8.8	13.5	16.4	17.8	19.2	19.0	19.4	19.7	18.6	17.6	15.5	7.4
sygl-1(BD mut)	8.4	13.7	16.5	18.6	19.4	20.2	20.0	19.7	19.5	17.7	16.3	8.1
sygl-1(CD mut)	8.4	12.5	16.0	16.7	17.9	18.7	19.0	18.4	17.7	17.3	15.8	7.8
sygl-1(ø)	9.0	13.6	15.7	17.8	18.4	18.1	18.1	16.9	16.6	14.8	13.9	6.9

B LBS effects on mRNA distribution: empty cells vs. cells containing *sygl-1* mRNA

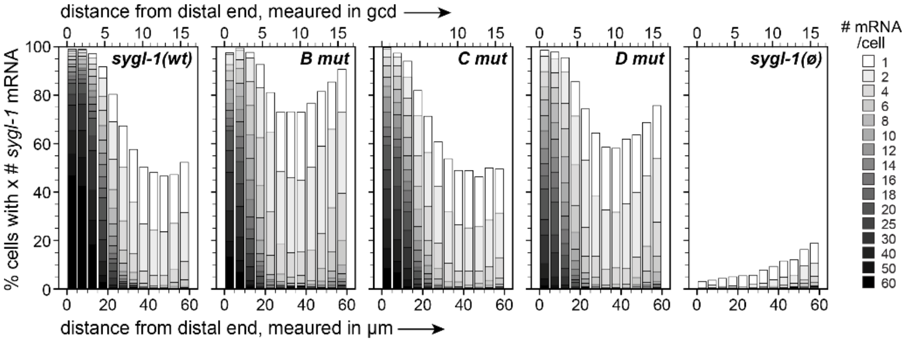

Fig. S4. Analyzing the cell-to-cell distribution of *sygl-1* mRNA in LBS mutants

A. Average number of nuclei per bin (region from the distal end). MATLAB reconstructs the gonad in 3D and assigns each nucleus center an x coordinate (Lee et al., 2016). Bin 1 corresponds to 0-5 µm from the distal end, bin 2 to 5-10 µm, and so on. The average nuclei/bin numbers were taken from the smFISH data set; see Fig. S2E for the total number of gonads scored. **B.** mRNA probability by position, shown as histograms. For each of 16 quantities of mRNA per cell (see legend on right), each value plotted is the single percentage calculated from pooled nuclear data and thus there are no error bars. The percentages of cells with ≥5 mRNAs in the 0-5 µm region are as follows: *wild-type(wt)*: 96.5%; *B mut*: 91.9%; *C mut*: 93.6%; *D mut*: 86.2%; *sygl-1(ø)*: 0%. The maximum numbers of mRNA/cell are as follows: *wild-type(wt)*: 146; *B mut*: 90; *C mut*: 111; *D mut*: 86; *sygl-1(ø)*: 3.

Development • Supplementary information

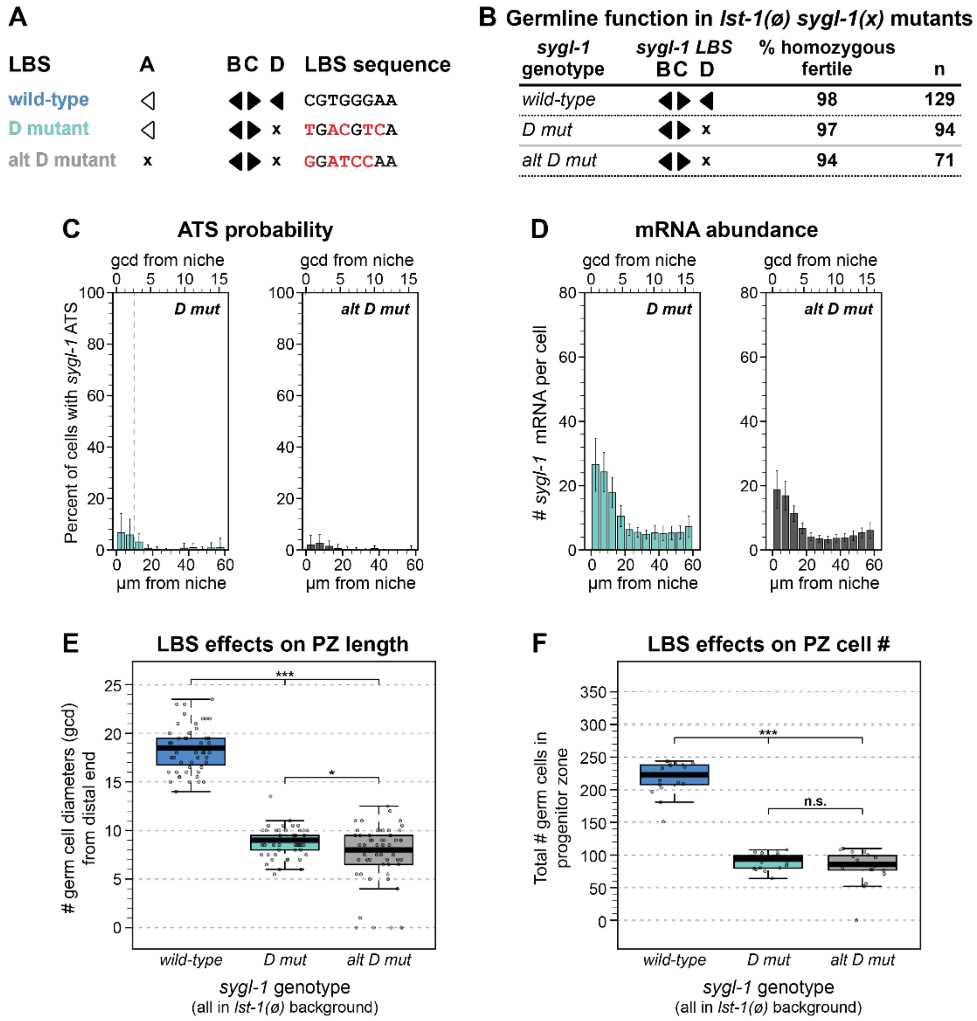

Fig. S5. An alternatively mutated LBS sequence, *alt D mut*, is homozygous fertile like other LBS singles but has marginal changes in *sygl-1* dose

A. Schematic of two distinct 5 bp LBS D mutations generated by Cas9 editing. Conventions as in Fig. 1E. Wild-type: 5’ CGTGGGAA 3’; *D mut*: 5’ TGACGTCA 3’; *altD mut*: 5’ GGATCCAA 3’ (differences from wild-type motif underlined). Unlike *D mut*, *alt D mut* mutates all three of the central guanines. In *alt D mut*, we mutated the middle G to a C: typically, the middle G is recognized as the most degenerate of the trio of guanines, but there is also *in vitro* evidence that suggests a mutation to C is particularly deleterious for CSL binding (Torella et al. 2014, Friedmann and Kovall 2010, others). **B-F.** *alt D mut* and *D mut* were directly compared. As in the rest of the paper, effects on molecular quantitation was scored in *lst-1(+)* (C-D) while effects on phenotype were scored in *lst-1(ø)* (B, E-F). **B.** Germline fertility was scored by presence of embryos in young adults, as in Fig. 4A. **C-D.** *alt D mut* (n = 56 gonads) was directly compared to *D mut* (n = 39 gonads) in 2 smFISH experiments. Because this figure only includes the *D mut* smFISH data that was directly compared to *alt D mut*, Fig. S5C,D graphs are a subset of the data shown in Figs. 2-3. **C.** Percentage of nuclei with ≥1 ATS as a function of distance. Conventions are as in Fig. 2C. **D.** Number of mRNA per cell as a function of distance from the distal end. Conventions are as in Fig. 3A. **E.** PZ length shown as boxplots, BoxPlotR conventions; center line: median. PZ observations from each gonad were fitted to a linear mixed effects model and Tukey’s post-hoc test was used to make pairwise comparisons between genotypes. ***: $p < 0.0001$; *: $p = 0.009$. Total gonads scored from two experiments: *wild-type:* 55; *alt D mut*: 57; *D mut*: 57. **F.** PZ cell number shown as boxplots, BoxplotR conventions. Automatic Imaris counts in DAO-5-stained gonads. ***: $p < 0.0001$; n.s.: $p = 0.43$. Total gonads scored: *wild-type*: 18; *alt D mut*: 20, *D mut*: 20 from one experiment.

Development • Supplementary information

A **Two versions of *sygl-1* null**

	LBS motifs intact?	*sygl-1* CDS present?	V5 tag on *sygl-1* C-term?
sygl-1(ø)	yes	no	no
sygl-1::V5(BCD mut)	no	yes	yes

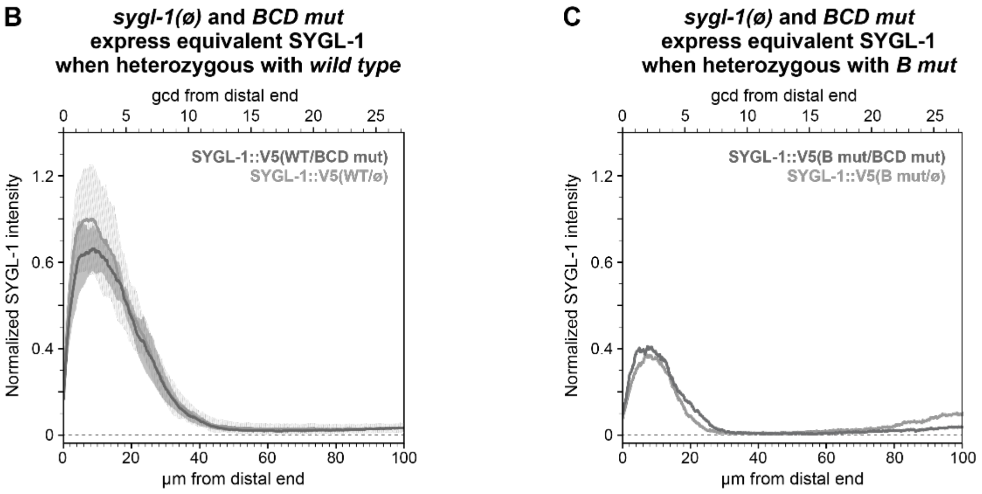

Fig. S6. Using *sygl-1(null)* strains in heterozygotes

A. Both *sygl-1(ø)* (Fig. 2A) and *sygl-1::V5(BCD mut)* (Fig. 1E) are null in the distal germline but differ in a few ways that made *BCD mut* a more suitable null allele for exploring LBS number and SYGL-1 dose. In *sygl-1(ø)*, Cas9 editing removed the *sygl-1* ORF from the start of the first exon to the end of the last exon but left UTRs and LBS intact. There is no V5 tag on *sygl-1(ø)*. In *BCD mut*, the *sygl-1* ORF is intact and V5-tagged, but all three LBSs are mutated. Neither allele expresses detectable SYGL-1 protein in the distal germline (Fig. 1F-G). **B-C.** *WT/null* or *B mut/null* heterozygotes produce similar results regardless of *sygl-1* null allele used. Quantification of α-V5 immunofluorescence as a function of distance from the distal end, conventions as in Fig. 3B. **B.** Total gonads scored: *WT/ø*: 24; *WT/BCD mut*: 30, from 2experiments each (*WT/ø* and *WT/BCD mut* immunostaining not done in parallel). **C.** Total gonads scored: 10 each for *B mut/ø* and *B mut/BCD mut*, immunostaining compared in parallel from one experiment.

Development • Supplementary information

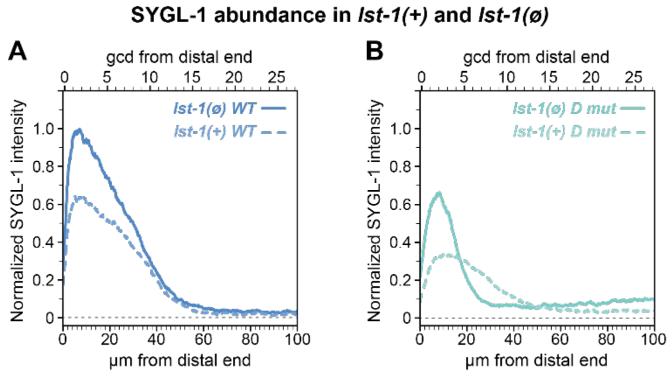

Fig. S7. Absence of *lst-1* increases SYGL-1 abundance

A-B. Quantification of α-V5 immunofluorescence. All four strains were assayed in parallel but separated into two graphs for clarity. 24 gonads/genotype scored in 2 replicates. Both graphs normalize SYGL-1 fluorescence intensity using the *lst-1(ø) sygl-1::V5(wild-type)* strain. The mechanism by which *lst-1* removal changes SYGL-1 abundance is not understood. However, understanding that SYGL-1 abundance is not the same in *lst-1(+)* and *lst-1(ø)* is relevant to this paper. **A.** *sygl-1::V5(wild-type)* abundance compared in *lst-1(+)* and *lst-1(ø)* backgrounds. **B.** *sygl-1::V5(D mut)* abundance compared in *lst-1(+)* and *lst-1(ø)* backgrounds.

Development • Supplementary information

Table S1. Strains used

Strain	Source	Description	Used in Figures
N2 bristol	Brenner, 1974	Wild-type	2B-F; 3A-F; 5A,D; S2A,D,E; S3; S4
JK4864 *qIs147*		*Psur5::GFP*	4E
JK5622 *sygl-1(q828) I*	Shin, 2017	*sygl-1(ø)*	1F-G; 2B,C,E; 3A,C,E; 5A,D; S1C; S2E; S3A; S4
JK5773 *sygl-1(q936) I*	this work	*sygl-1(LBS C mut)*	2C-F; 3A,C; S2B,D,E; S3A; S4
JK5796 *lst-1(q869) I/hT2[qIs48](I;III)*	Shin, 2017	*lst-1(ø)*	4A,C,D; 6A-B; S5B,E,F; S7
JK5812 *sygl-1(q942) I*	this work	*sygl-1(LBS CD mut)*	2C-F; 3E; 5A,D; S2C-E; S3A; S4A
JK5813 *sygl-1(q943) I*	this work	*sygl-1(LBS D mut)*	2B-F; 3A,C; S2B,D,E; S3A; S4; S5C,D
JK5911 *lst-1(q869) sygl-1(q942) I/hT2[qIs48](I;III)*	this work	*lst-1(ø) sygl-1(LBS CD mut)*	4A; 5B
JK5912 *sygl-1(q956) I*	this work	*sygl-1(LBS B mut)*	2C-F; 3A,C; S2B,D,E; S3A; S4
JK6002 *sygl-1(q1015) I*	Shin, 2017	*sygl-1::1xV5(wild-type)*	1F-G; 3B,D,F; S1C,E; S3B-C; S7
JK6020 *lst-1(q869) sygl-1(q943) I/hT2[qIs48](I;III)*	this work	*lst-1(ø) sygl-1(LBS D mut)*	4A,C,D; S5B,E-F
JK6065 *sygl-1(q1039) I*	this work	*sygl-1(LBS BD mut)*	2C-F; 3E; 5A,D; S2C-E; S3A; S4A
JK6111 *sygl-1(q1054) I*	this work	*sygl-1::1xV5(LBS D mut)*	3B-C; S3B; S7
JK6161 *sygl-1(q1101) I*	this work	*sygl-1(LBS BC mut)*	2B-F; 3E; 5A,D; S2C-E; S3A; S4A
JK6165 *lst-1(q869) sygl-1(q1039) I/hT2[qIs48](I;III)*	this work	*lst-1(ø) sygl-1(LBS BD mut)*	4A; 5B
JK6180 *lst-1(q869) sygl-1(q1101) I/hT2[qIs48](I;III)*	this work	*lst-1(ø) sygl-1(LBS BC mut)*	4A; 5B
JK6192 *sygl-1(q1116) I*	this work	*sygl-1(LBS alt D mut)*	S5C,D
JK6219 *lst-1(q869) sygl-1(q936) I/hT2[qIs48](I;III)*	this work	*lst-1(ø) sygl-1(LBS C mut)*	4A,C,D
JK6288 *sygl-1(q1135) I*	this work	*sygl-1::1xV5(LBS BC mut)*	3F; S3C
JK6289 *sygl-1(q1136) I*	this work	*sygl-1::1xV5(LBS BD mut)*	3F; S3C
JK6387 *sygl-1(q1163) I*	this work	*sygl-1::1xV5(LBS A mut)*	1F-G; S1C
JK6388 *sygl-1(q1165) I*	this work	*sygl-1::1xV5(LBS C mut)*	3B,D; S3B
JK6389 *sygl-1(q1167) I*	this work	*sygl-1::1xV5(LBS BCD mut)*	1F-G; S1B-C
JK6390 *lst-1(q869) sygl-1(q956) I/hT2[qIs48](I;III)*	this work	*lst-1(ø) sygl-1(LBS B mut)*	4A,C,D
JK6391 *lst-1(q869) sygl-1(q1116) I/hT2[qIs48](I;III)*	this work	*lst-1(ø) sygl-1(LBS alt D mut)*	S5B,E-F

JK6401 *lst-1(q869) sygl-1(q828) I hT2[qIs48](I;III)*	this work	*lst-1(ø) sygl-1(ø)*	5B; S6B-C
JK6431 *lst-1(q869) sygl-1(q1015) I/ hT2[qIs48](I;III)*	this work	*lst-1(ø) sygl-1::1xV5(wild-type)*	4E; 6A-C; S6B-C; S7
JK6507 *lst-1(q869) sygl-1(q1136) I/ hT2[qIs48](I;III)*	this work	*lst-1(ø) sygl-1::1xV5(BD mut)*	6D-E
JK6508 *sygl-1(q1231) I*	this work	*sygl-1::1xV5(LBS B mut)*	3B,D; S3B
JK6516 *lst-1(q869) sygl-1(q1054) I/hT2[qIs48](I;III)*	this work	*lst-1(ø) sygl-1::1xV5(LBS D mut)*	4E; 6A; S7
JK6517 *lst-1(q869) sygl-1(q1231) I/ hT2[qIs48](I;III)*	this work	*lst-1(ø) sygl-1::V5(B mut)*	6B-E; S6C
JK6521 *lst-1(q869) sygl-1(q1015) I; emb-30(tn377) III/hT2[qIs48](I;III)*	this work	*lst-1(ø) sygl-1::1xV5(wild-type); emb-30*	4F-G
JK6522 *sygl-1(q1220) I*	this work	*sygl-1::1xV5(LBS A canonical)*	S1E
JK6539 *lst-1(q869) sygl-1(q1136) I/hT2[qIs48](I;III); rol-6(e187)*	this work	*lst-1(ø) sygl-1::1xV5(LBS BD mut); rol-6*	6D-E
JK6566 *sygl-1(q1253) I*	this work	*sygl-1::1xV5(LBS CD mut)*	3E; S3C
JK6567 *lst-1(q869) sygl-1(q1054) I; emb-30(tn377) III/hT2[qIs48](I;III)*	this work	*lst-1(ø) sygl-1::1xV5(LBS D mut); emb-30*	4F-G
JK6600 *lst-1(q869) sygl-1(q1167) I/hT2[qIs48](I;III)*	this work	*lst-1(ø) sygl-1::1xV5(BCD mut)*	6B-E; S1B; S6

*all **1xV5** strains also include a GGS linker (**ggtaagcctatccctaaccctctcctcggtctAgatAGTacT**GGAGGATCC)

Table S2. Custom DNA probes for sygl-1 smFISH

Click here to download Table S2
